# Lef1 regulates caveolin expression and caveolin dependent endocytosis, a process necessary for Wnt5a/Ror2 signaling during *Xenopus* gastrulation

**DOI:** 10.1038/s41598-019-52218-1

**Published:** 2019-10-30

**Authors:** Katharina Puzik, Veronika Tonnier, Isabell Opper, Antonia Eckert, Lu Zhou, Marie-Claire Kratzer, Ferdinand le Noble, Gerd Ulrich Nienhaus, Dietmar Gradl

**Affiliations:** 1 Department of Cell and Developmental Biology, Karlsruhe Institute of Technology, 76128 Karlsruhe, Germany; 2 Institute of Applied Physics, Karlsruhe Institute of Technology, 76128 Karlsruhe, Germany; 3 Institute of Toxicology and Genetics, Karlsruhe Institute of Technology, 76344 Eggenstein-Leopoldshafen, Germany; 4 Institute of Nanotechnology, Karlsruhe Institute of Technology, 76344 Eggenstein-Leopoldshafen, Germany; 5 Department of Physics, University of Illinois at Urbana-Champaign, Urbana, Illinois 61801 USA

**Keywords:** Cell biology, Developmental biology

## Abstract

The activation of distinct branches of the Wnt signaling network is essential for regulating early vertebrate development. Activation of the canonical Wnt/β-catenin pathway stimulates expression of β-catenin-Lef/Tcf regulated Wnt target genes and a regulatory network giving rise to the formation of the Spemann organizer. Non-canonical pathways, by contrast, mainly regulate cell polarization and migration, in particular convergent extension movements of the trunk mesoderm during gastrulation. By transcriptome analyses, we found caveolin1, caveolin3 and cavin1 to be regulated by Lef1 in the involuting mesoderm of *Xenopus* embryos at gastrula stages. We show that caveolins and caveolin dependent endocytosis are necessary for proper gastrulation, most likely by interfering with Wnt5a/Ror2 signaling. Wnt5a regulates the subcellular localization of receptor complexes, including Ror2 homodimers, Ror2/Fzd7 and Ror2/dsh heterodimers in an endocytosis dependent manner. Live-cell imaging revealed endocytosis of Ror2/caveolin1 complexes. In *Xenopus* explants, in the presence of Wnt5a, these receptor clusters remain stable exclusively at the basolateral side, suggesting that endocytosis of non-canonical Wnt/receptor complexes preferentially takes place at the apical membrane. In support of this blocking endocytosis with inhibitors prevents the effects of Wnt5a. Thus, target genes of Lef1 interfere with Wnt5a/Ror2 signaling to coordinate gastrulation movements.

## Introduction

Different branches of the Wnt signaling network, including the Wnt/β-catenin and the β-catenin independent branches are tightly regulated to ensure proper cell differentiation and dynamics during early embryogenesis^1^. Activation of the canonical Wnt/β-catenin signaling pathway results in stabilization of β-catenin in the cytoplasm, which subsequently translocates into the nucleus^2^. There, it binds to transcription factors of the Tcf/Lef family and regulates expression of numerous target genes, which are essential for the formation of the dorso-ventral axis^3^. Thus, the Wnt/β-catenin branch is required prior to gastrulation for induction and positioning of the Spemann organizer.

Consistently, knock-down of Tcf1 and Tcf4 impairs Wnt/β-catenin signaling in the mesoderm^4,5^ and central nervous system^6,7^ of developing *Xenopus* embryos. Interestingly, knock-down of Lef1 additionally impairs convergent extension (CE) movements at gastrula stages^8^. Thus, in addition to the β-catenin independent Wnt/Ca^++^ ^8^, Wnt/PCP^9,10^ and Wnt/Ror2^11^ branches, a transcription factor of the Wnt/β-catenin pathway is essential for the regulation of these collective tissue movements.

Lef1 regulates the expression of Wnt/β-catenin target genes. Among them, *Xnr-3* has been shown to be relevant for CE movements^8^. Additional target genes might also be important, however, to provide the involuting mesoderm with the competence to react to non-canonical Wnt signaling to execute gastrulation movements. These β-catenin independent Wnt signaling pathways execute their function mainly *via* regulation of cell shape and behavior. At early gastrulation stages, Wnt11 triggers multipolar dorsal mesodermal cells to become bipolar^12^. Wnt5a, however, is responsible for migration of the polarized cells towards the dorsal midline. Polarization and migration result in a medio-lateral intercalation of axial mesodermal cells, causing medio-lateral narrowing (convergence) and anterior-posterior elongation (extension) of the dorsal trunk mesoderm^11,12^. Therefore, Wnt5a and Wnt11 are the main regulators of CE movements.

In light of the many levels of crosstalk between Wnt/β-catenin and non-canonical Wnt signaling^8,13^, we asked whether Lef1 regulates CE movements by influencing the expression of non-canonical Wnt network components. However, in a transcriptome analysis of dorsal marginal zone (DMZ) explants we did not find core components of non-canonical Wnt signaling among the numerous putative Lef1 target genes. Instead, we identified *caveolin1*, *caveolin3* and *cavin1* (polymerase I and transcript release factor, PTRF) as Lef1 regulated genes. Caveolin and cavin proteins form the structural core of caveolae. These cholesterol rich membrane invaginations become internalized and traffic as caveosomes along recycling pathways or end up in multivesicular bodies or lysosomes^14,15^. These findings prompted us to investigate whether non-canonical branches of the Wnt network are regulated by caveolin dependent endocytosis.

So far it was shown that the activity of the canonical Wnt/β-catenin branch is regulated by endocytosis in at least two ways. On the one hand, dkk1/lrp complexes are internalized in a clathrin dependent manner, resulting in a decrease of membrane bound lrp and thus down-regulation of Wnt/β-catenin signaling^16,17^. On the other hand, Wnt signalosomes are internalized in a caveolin dependent manner, resulting in a depletion of the cytosolic GSK3β pool, accumulation of β-catenin and activation of Wnt/β-catenin signaling^16,17^. The regulation of Wnt/β-catenin signaling, and also the regulation of other signaling pathways including fgf^18,19^ and vegf^20,21^ signaling, together with many other functions ascribed to caveolae and caveosomes implicate an important role of these subcellular structures for embryogenesis. However, cav1/cav3 double knock-out mice lacking any detectable caveolae are viable^22^, which suggests that mechanisms are activated in early mouse embryogenesis that can compensate for the loss of caveolae.

Here we show that a controlled expression of caveolar core components and caveolin dependent endocytosis are essential for proper gastrulation movements during early *Xenopus* development. Epistasis experiments revealed that these caveolae core components cooperate with Wnt5a in regulating CE movements. Wnt5a induces clustering of Ror2 receptor complexes, consisting of Ror2, Fzd7 and Disheveled (dsh). These receptor complexes are internalized both in a caveolin and clathrin dependent manner. In polarized cells of the animal ectoderm, internalization occurs predominantly at the apical surface. We provide evidence that internalization is essential for full activation of the Wnt5a response.

## Results

**Figure 1:**
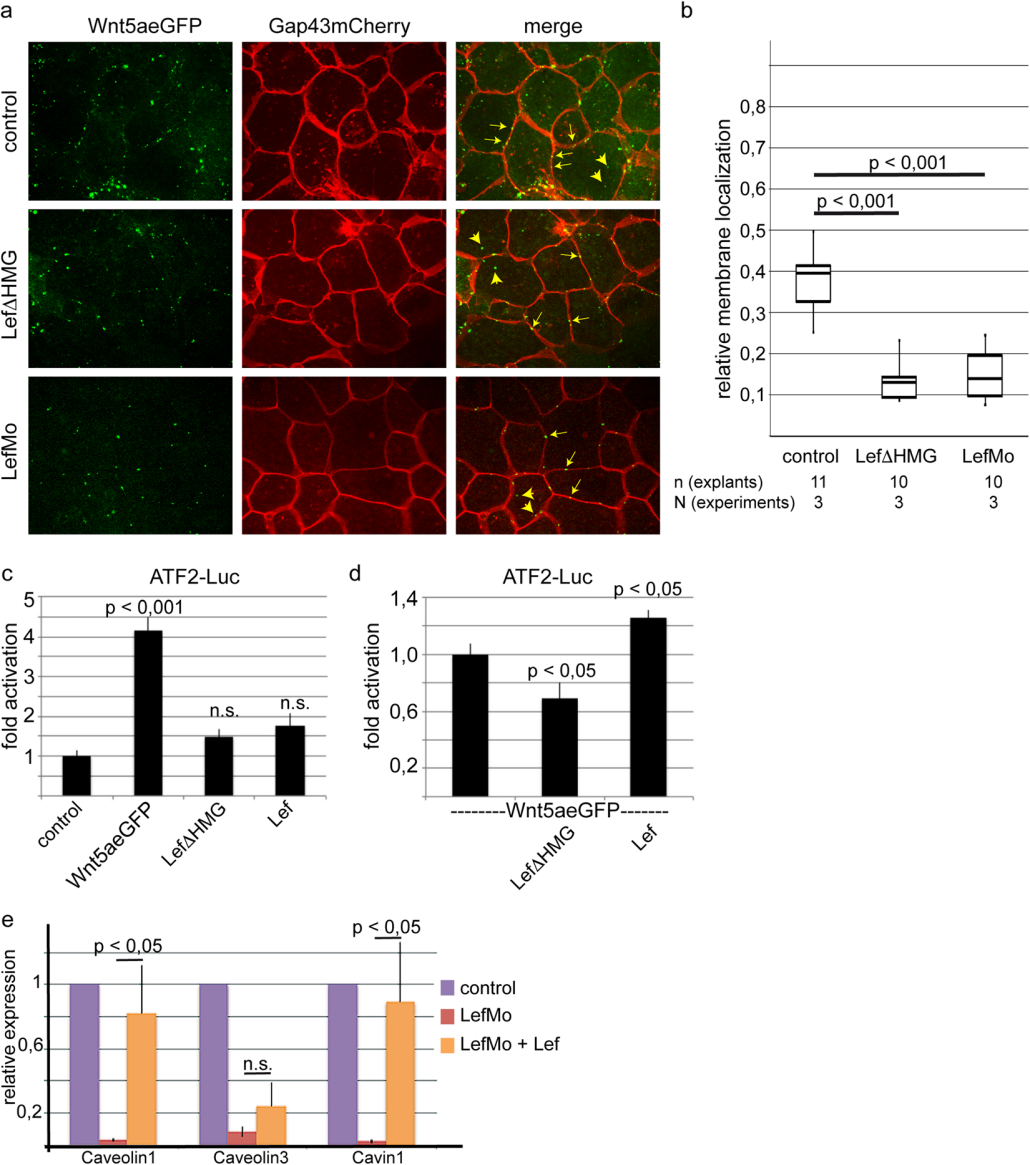
Lef1 regulates Wnt5a clustering and the expression of caveolin family members. **(a**) Knock-down of Lef1 reduces the formation of Wnt5a clusters at the plasma membrane of DMZ cells. Both dorsal blastomeres of *Xenopus* 4-cell stage embryos were injected with 150 pg Wnt5aeGFP mRNA together with the membrane marker GAP43mCherry and 1 pmol of the Lef1 specific antisense morpholinos (LefMo) or 500 pg dominant negative Lef1 mRNA (Lef∆HMG). The arrows indicate some Wnt5a clusters located at the plasma membrane, arrowheads point to cytoplasmically located Wnt5a clusters. (**b**) Downregulation of Lef1 reduces the presence of Wnt5a clusters at the plasma membrane. Shown are the relative membrane localization according to Manders *et al*.^53^ and the p values according to Student’s t-test. The images were processed using the Coloc 2 plugin in ImageJ which allows calculation of the Manders coefficient M2^53^. This coefficient indicates colocalization of Wnt5aeGFP clusters and the red coloured membrane. (**c**) In transfected HEK293 cells, Lef1∆HMG and wild-type Lef1 do not significantly regulate the non-canonical Wnt reporter ATF2Luc. (**d**) In Wnt5a stimulated cells (cotransfected with Wnt5aeGFP), Lef1∆HMG represses ATF2Luc activity. Wild-type Lef1, instead, activates the ATF2Luc reporter. p values of at least nine independent transfections were determined according to Student’s t-test. e) Real-time RT-PCR confirmed that the expression of cavin1, caveolin1 and caveolin3 are downregulated in Lef1 morphants and partially restored in morphants co-injected with 500 pg Lef1 mRNA. Shown are mean values and standard error of four biological replicates.

We have recently observed Wnt5a clustering together with Ror2 in the plasma membrane of dorsal marginal zone explants^23^. Here we show that Wnt5a cluster formation depends on the presence of Lef1. The Wnt5aeGFP clusters at the Gap43-mCherry marked membrane were determined by colocalization analysis using Manders coefficient M2. Due to the correlation with the membrane we can distinguish between membrane and non-membrane Wnt5aeGFP clusters. Interestingly, the proportion of Wnt5a clusters located in the membrane decreases in Lef1 morphants and Lef∆HMG expressing DMZ explants (Fig. 1a,b), indicating that both, Wnt5a cluster formation and localization, depend on Lef1 and thus on Lef1 regulated target gene expression. Furthermore, in transient transfectants, Lef1 hyperactivates the promoter in Wnt5a overexpressing cells, whereas dominant negative Lef1 impairs Wnt5a driven ATF2Luc activation (Fig. 1c,d). This led us to hypothesize that Lef1 regulated gene expression interferes with non-canonical Wnt signaling.

In order to identify Lef1 target genes relevant for regulating non-canonical Wnt signaling we performed an ATLAS microarray-based transcriptome analysis and compared the expression profiles of DMZ explants derived from Lef1 morphants (LefMo) and control morpholino injected embryos (Supplementary Fig. 1a). Among the TOP100 differentially regulated genes (Supplementary Table 1) we found to our surprise that *caveolin1*, *caveolin3* and *cavin1*, three core components of caveolae, were significantly down-regulated. This result was further confirmed by independent Real-time PCR experiments, showing that the expression of cavin1, caveolin1 and caveolin3, was down-regulated in Lef1 morphants and partially restored by co-expressed Lef1 (Fig. 1e).

We amplified the open reading frames of *caveolin1*, *caveolin3* and *cavin1* from cDNA of DMZ explants and analyzed their expression pattern by *in situ* hybridization. At gastrula stages, *cavin1* and *caveolin1* appear enriched at the dorsal blastopore. From early to late neurula stages, both genes are expressed in the notochord. This expression in the axial mesoderm is still highly enriched at tailbud stages (Supplementary Fig. 1b). *Caveolin3* instead becomes localized in a salt and pepper pattern from early tailbud stages onward (Supplementary Fig. 1b). This allowed us to confirm the Lef1 dependency in unilaterally injected embryos (Supplementary Fig. 1c). Indeed, *cav3* expression was reduced in Lef1 morphants and co-injected Lef1 restored *cav3* expression (Supplementary Fig. 1c).

We did not identify non-canonical Wnt signaling components among the Lef1 target genes (TOP100 list in Supplementary Table 1, complete list available at GEO GSE84488). However, the identification of three genes encoding for caveolar core components raises the question whether these proteins are required for CE movements and if non-canonical Wnt signaling depends on caveolae.

### Caveolin dependent internalization is required for proper gastrulation movements

**Figure 2:**
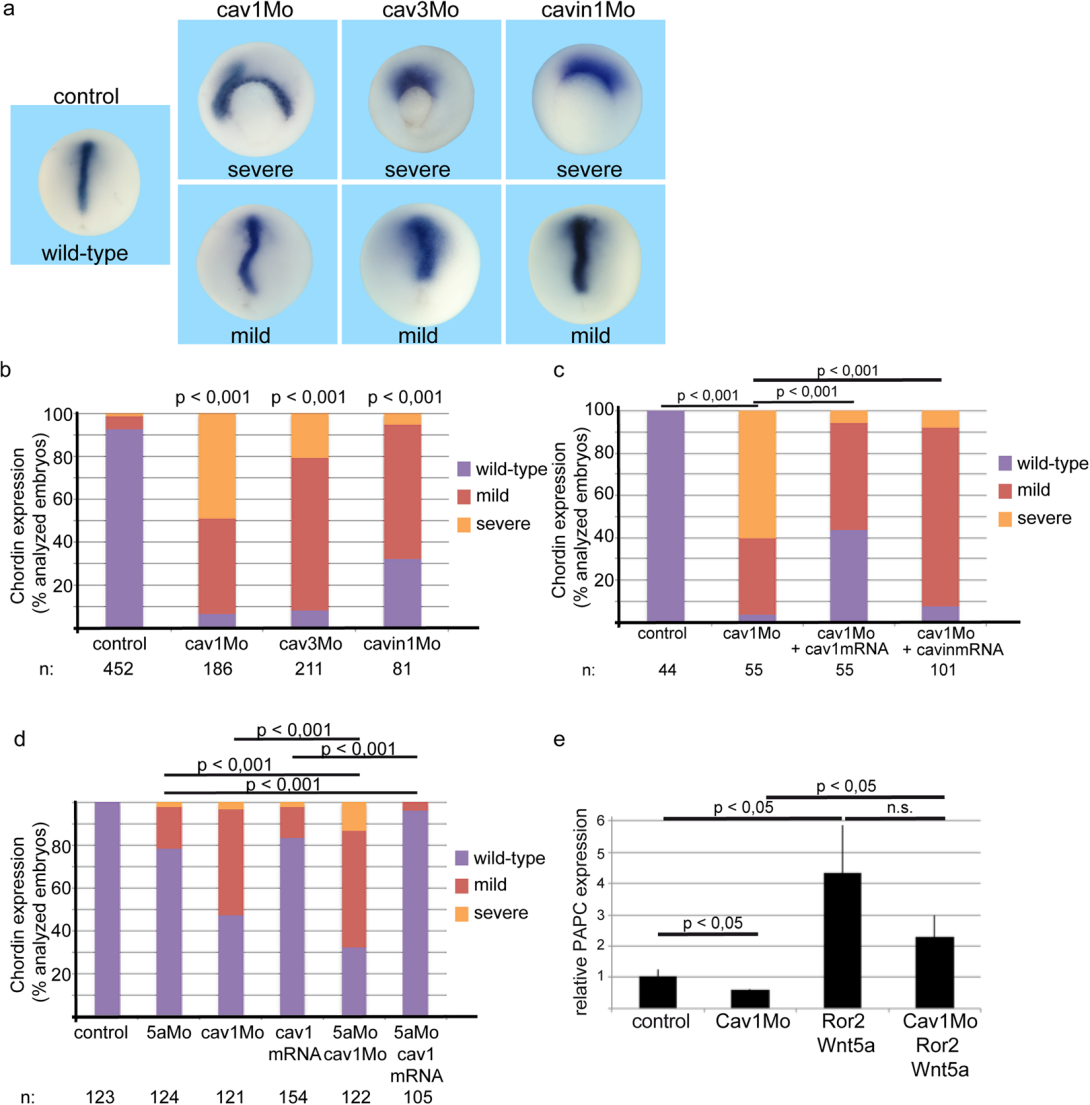
The caveolar core components caveolin1, caveolin3 and cavin1 are necessary for convergent extension movements. Antisense morpholino oligonucleotides directed against caveolin1 (cav1Mo), caveolin3 (cav3Mo) and cavin1 (cavin1Mo) were injected in the marginal zone of both dorsal blastomeres of 4-cell stage embryos. Embryos were fixed at stage 14 and analyzed for localization of the axial mesodermal marker gene *chordin*. (**a**) In control embryos, *chordin* is expressed in a long and narrow strip along the dorsal midline. Depletion of caveolar proteins resulted in mislocalization of *chordin* expression. In severe cases, *chordin* expression remained at the dorsal part of the unclosed blastopore. The phenotype was classified as mild when the *chordin* expression was bent or short and broad. (**b**) Quantification of the phenotypes in the morphants resulting from the injection of 4 pmol cav1Mo, 4 pmol cav3Mo and 0.4 pmol cavin1Mo, respectively. (**c**) Caveolin1 and cavin1 patially restore chordin expression in cav1 morphants. 4 pmol cav1Mo were coinjected with 500 pg caveolin1 and cavin1 mRNA, respectively. (**d**) Epistasis experiments with Wnt5a morpholino (5aMo, 0.4 pmol), caveolin1 morpholino (cav1Mo, 0.25 pmol) and caveolin1 mRNA (500 pg) revealed additive effects of the morpholinos. Furthermore, caveolin1 mRNA partially restored gastrulation in Wnt5a morphants. P values according to a χ^2^ test, n: number of embryos. (**e**) Realtime PCR of animal cap explants revealed that the expression of the Wnt5a target gene *PAPC* depends on caveolin1 expression. Shown are the mean values, standard errors and p values according to Student’s t-test of four independent experiments.

We knocked down caveolin1, caveolin3 and cavin1 (Supplementary Fig. 2a) and investigated whether CE movements depend on the presence of these proteins. Indeed, morpholino knock-down of caveolin1, caveolin3 and cavin1 resulted in gastrulation defects (Fig. 2a,b). For quantification of these effects, we classified the phenotypes into three categories: wild-type embryos show a long and narrow strip of *chordin* expression indicating wild-type elongation of the axial mesoderm and thus wild-type CE movements. The mild phenotype is defined by a shortened and broad *chordin* expression or bends in the axial mesoderm. The severe phenotype is characterized by *chordin* expression stacking at the dorsal site of the non-closing blastoporus (Fig. 2a). Interestingly, knock-down of each of the caveolar core components resulted in mislocalization of *chordin* (Fig. 2a,b), indicating that caveolae are involved in the regulation of CE movements. The frequency and severity of the phenotype depends on the morpholino dose (Supplementary Fig. 2b). At high doses, the cavin1 morpholino is toxic and the embryos die during gastrulation. In rescue experiments overexpressed caveolin1 and cavin 1 mRNA can compensate for the loss of caveolin1 (Fig. 2c) and for the loss of cavin1 (Supplementary Fig. 2c). Taken together, expression of caveolin1, caveolin3 and cavin1 is essential for proper gastrulation.

In epistasis experiments, we injected caveolin1 mRNA and caveolin1 morpholino into Wnt5a morphants. Simultanous knock-down of Wnt5a and caveolin1 induced more severe mislocalization of *chordin* than single morpholinos. Furthermore, overexpressed caveolin1 partially compensated for the loss of Wnt5a (Fig. 2d), suggesting that Wnt5a and caveolin1 are part of one pathway. This result prompted us to examine whether caveolins might regulate non-canonical Wnt signal transduction. As a first coarse experiment we tested if caveolin1 and caveolin3 regulate the non-canonical Wnt reporter ATF2Luc in transiently transfected HEK293 cells. Both, overexpressed caveolin1 and caveolin3 slightly increased ATF2Luc activity (Supplementary Fig. 2d), indicating that caveolae are involved in Wnt5a triggered signal transduction. Real-time RT-PCR in animal cap tissue revealed that Wnt5a/Ror2 dependent regulation of *PAPC* partially depends on caveolin1. Co-injected Wnt5a and Ror2 induce *PAPC* expression; in cav1 morphants *PAPC* activation is much less apparent (Fig. 2e). However, overexpression of caveolins cannot compensate the loss of Lef1 (Supplementary Fig. 2f), indicating that other Lef1 target genes must also play a role for CE movements.

**Figure 3:**
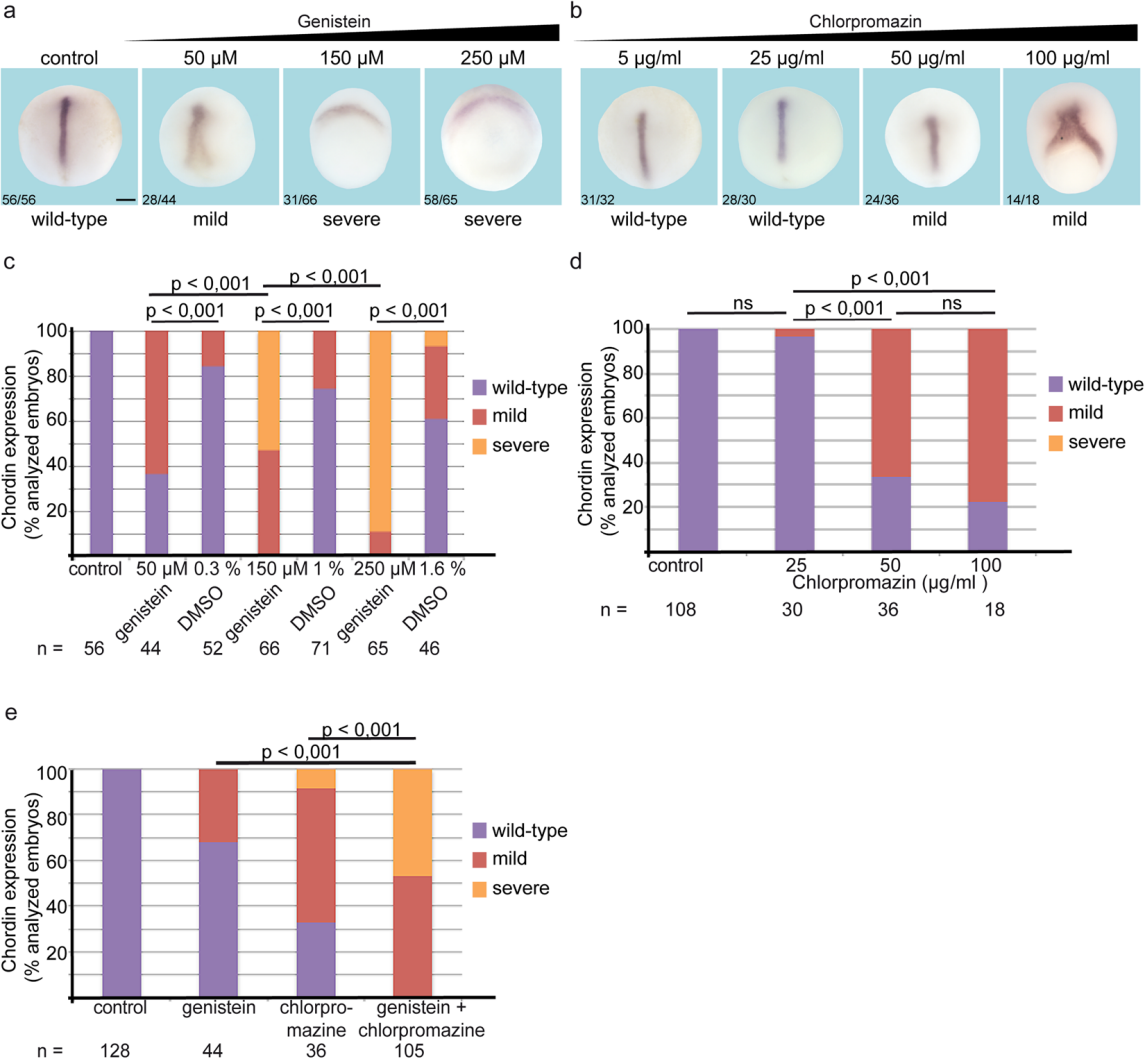
Endocytosis is necessary for convergent extension movements. Wild-type embryos were incubated from stage 9 to stage 14 in MBSH containing the indicated concentration of genistein (**a**) or chlorpromazine (**b**). (**c**) Quantification of the *chordin* expression revealed that genistein blocked convergent extension movements even at low doses (50 µM). At high doses (250 µM), almost all embryos showed the severe phenotype with *chordin* being expressed at the dorsal side of the open blastoporus, whereas 60% of the DMSO-treated siblings expressed *chordin* in a wild-type pattern. (**d**) Quantification of the *chordin* expression revealed that 50 µg/ml chlorpromazine induced a mild mislocalization of *chordin* expression in more than 60% of the embryos. Doubling of the dose to 100 µg/ml increased the frequency of *chordin* mislocalization to more than 70%. e) Simultaneous treatment with both inhibitors revealed additive effects. Quantification of *chordin* mislocalization revealed that both inhibitors together inhibit convergent extension movements even at doses when one inhibitor alone has only mild effects. n: number of embryos, p-values according to χ^2^ test. n.s. not significant,

Our results showing that CE and Wnt5a signal transduction depend on caveolae raise the question if caveolin dependent endocytosis is also important in this context. Therefore, we blocked caveolae dependent endocytosis with the inhibitor genistein. In parallel, we inhibited clathrin dependent endocytosis with the inhibitor chlorpromazine. Both inhibitors impaired gastrulation movements in a dose dependent manner, as shown by the mislocalization of the axial mesodermal marker gene *chordin* (Fig. 3a–d). To test for compensatory mechanisms, we applied both inhibitors simultaneously and, indeed, CE was efficiently blocked (Fig. 3e). Furthermore, if we first treated the embryos with low doses of genistein and subsequently added low doses of chlorpromazine, the embryos failed to gastrulate properly (not shown). These results indicate that both clathrin and caveolin dependent endocytosis pathways regulate CE in a partially redundant manner.

To identify time windows during which caveolin-dependent endocytosis is relevant for CE movements, we added and washed off genistein at different time points from late blastula stages (stage 9) until late gastrula (stage 12) and early neurula stages (stage 14). Gastrulation movements were only efficiently blocked if the inhibitor was present throughout gastrulation, from stage 9 until stage 12. Adding the inhibitor after onset of gastrulation (stage 10) or washing it off before blastopore closure (stage 11) did not impair blastopore closure or *chordin* localization (Supplementary Fig. 3a–c). These findings indicate that caveolin dependent endocytosis is not only involved in bottle cell formation or polarization of the microtubule cytoskeleton at the onset of gastrulation, but is required during the entire gastrulation process. Moreover, the longer caveolin dependent endocytosis is blocked, the more severe are the malformations of the embryos.

Furthermore, in tissue culture cells, expression of *pbk*, a target gene negatively regulated by Wnt5a^24^ is upregulated in genistein-treated cells, even in the presence of co-transfected Ror2 and Wnt5a (Supplementary Fig. 3d), indicating that also this Wnt5a/Ror2 target gene is regulated *via* caveolin dependent endocytosis.

So far, our data revealed that the Lef1 regulated genes caveolin1, caveolin3 and cavin1 are important for CE movements, that endocytosis is relevant and, that caveolins and endocytosis are involved in regulating Wnt5a/Ror2 signal transduction.

### Wnt5a induces clustering and internalization of receptor complexes

We then analyzed Wnt induced cluster formation of the Wnt5a receptor Ror2 to explore how caveolins regulate non-canonical Wnt signaling. We have recently shown that Wnt5a recruits Ror2 into Wnt-positive clusters at the membrane of DMZ explants^23^. Receptor clustering seems to be an immediate response to Wnt5a, since Ror2 clusters are formed within a few minutes after adding commercially available Wnt5a protein (Supplementary Fig. 4, Supplementary Movie 1).

To analyze if the Ror2 clusters consist of individually arranged Ror2 monomers or Ror2/Ror2 dimers, and to analyze whether Ror2/Ror2 dimers form a distinct, Wnt5a induced sub-population of receptor molecules, we used the bimolecular fluorescence complementation (BiFC) assay (split-YFP assay). mRNA of the Ror2YN- and Ror2YC-constructs were co-injected into the animal half of *Xenopus* two-cell stage embryos. Live imaging of animal cap cells from these injected embryos revealed that, in the absence of Wnt5a, Ror2 homodimers and Ror2/Fzd7 heterodimers were uniformly distributed along the entire plasma membrane (Supplementary Fig. 5a). The cysteine rich extracellular domain (CRD) of Ror2 is believed to be the binding site for the Wnt ligand^25^. Here we show that the CRD is essential for Ror2 homodimerization. Ror2∆CRD constructs do not form homodimers (Supplementary Fig. 5a).

**Figure 4:**
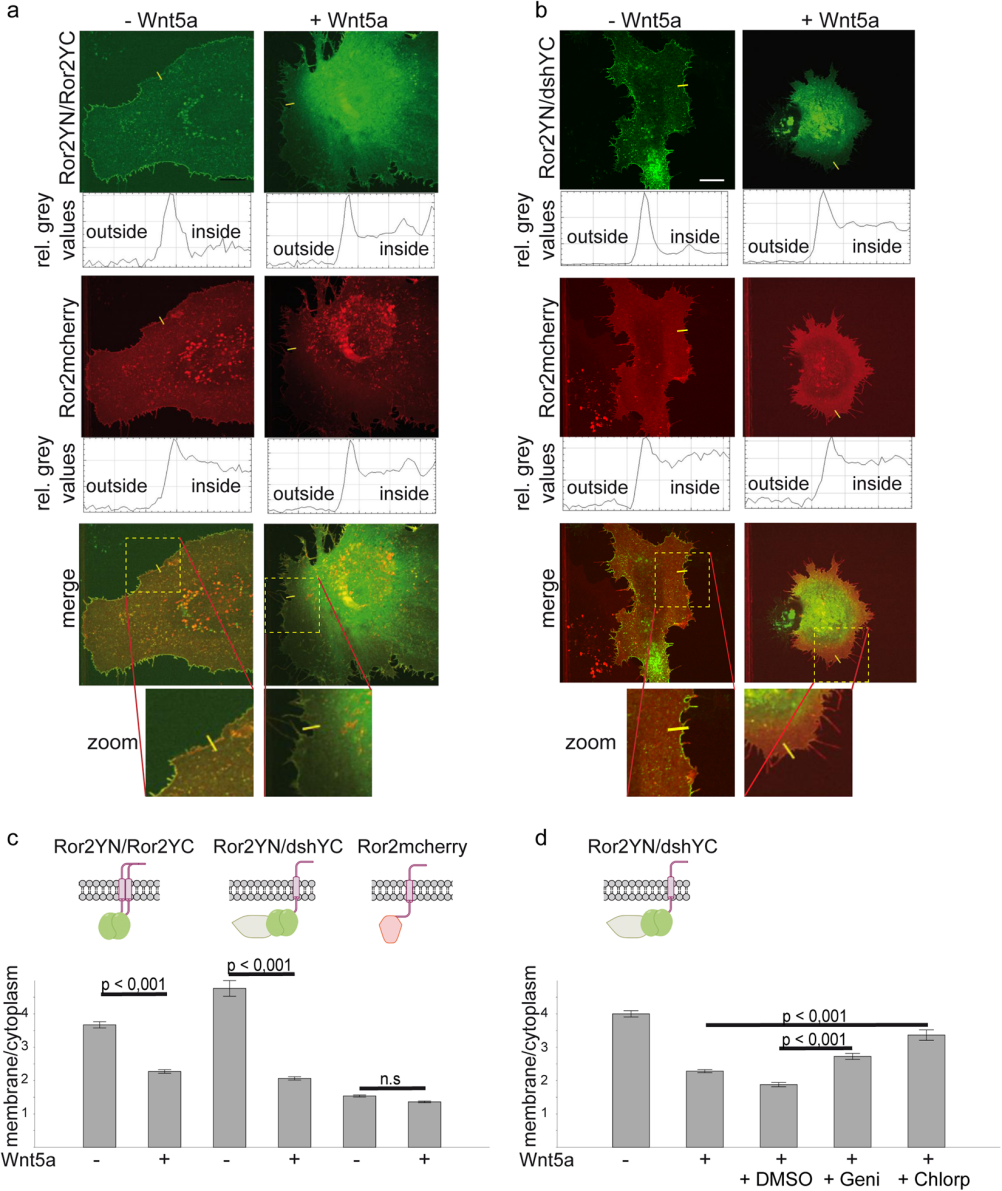
Wnt5a induces redistribution of Ror2 homodimers and Ror2/dsh heterodimers. The subcellular localization of fluorophore tagged Ror2 homodimers (**a**) and Ror2/dsh heterodimers (**b**) was analyzed by a bimolecular fluorescence complementation assay (BiFC) in transiently transfected *Xenopus* tissue culture cells. The relative grey values show the intensity profiles at the positions marked by yellow bars. (**c**,**d**) The membrane/cytoplasm values were calculated in the following way: membrane/cytoplasm = (intensity at the membrane – intensity outside)/(intensity inside – intensity outside). Shown are the mean values and standard errors of at least 399 profiles of more than 60 cells from at least ten independent experiments. Median and dispersion (25–75% range) are shown as box-and-whiskers plots in Supplementary Fig. 5b. (**d**) The effect of endocytosis inhibitors chlorpromazine and genistein on the subcellular distribution of Ror2/dsh heterodimers. Shown are the mean values and standard errors of at least 280 profiles of more than 40 cells in at least seven independent experiments. p values according to Student’s t-test, n.s.: not significant. Median and dispersion are shown in Supplementary Fig. 5c.

In transfected *Xenopus* tissue culture cells, we observed that the membrane fraction of Ror2 homodimers decreased with respect to the cytosolic fraction upon co-transfection with Wnt5a (Fig. 4a,c). Similarly, Ror2/dsh heterodimers were also translocated from the membrane to the cytoplasm in Wnt5a co-transfectants (Fig. 4b,c). Interestingly, the effect is much less pronounced for mCherry-tagged Ror2, which labels all Ror2 populations including monomers (Fig. 4a–c, Supplementary Fig. 5b). This indicates that only parts of the membrane located Ror2 molecules are bound in dimers. Thus, Wnt5a induces redistribution of Ror2/Ror2 and Ror2/dsh complexes with little or no effect on monomeric Ror2 molecules. To analyze whether endocytosis is responsible for Ror2/dsh redistribution, we added the clathrin inhibitor chlorpromazine and the caveolin inhibitor genistein. Indeed, both inhibitors impaired Wnt5a induced redistribution of Ror2/dsh heterodimers, indicating that Wnt5a induces clathrin and caveolin dependent internalization of Ror2 clusters (Fig. 4d and Supplementary Fig. 5c).

**Figure 5:**
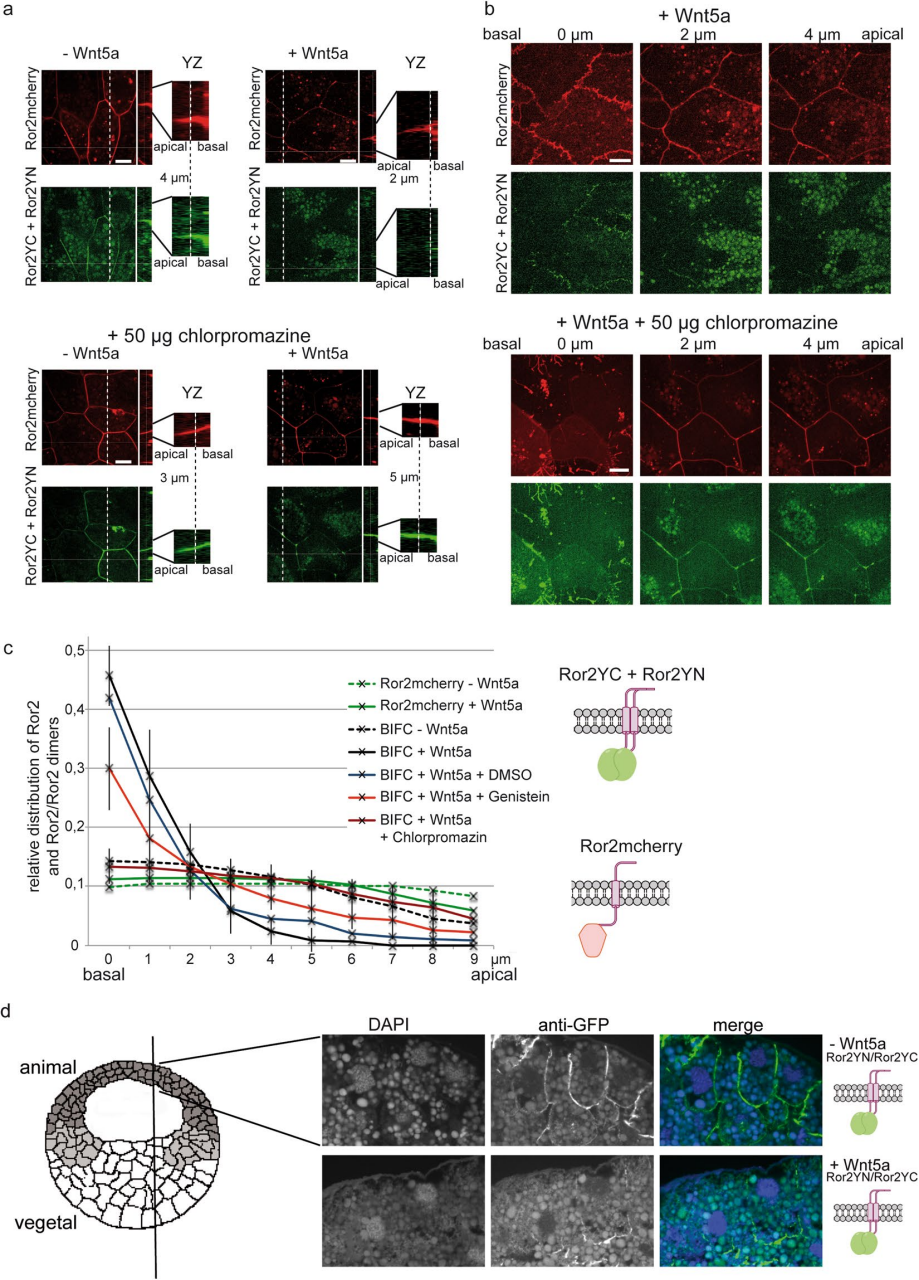
Wnt5a induces disappearance of Ror2/Ror2 dimers at the apical membrane. (**a**) Animal cap explants were analyzed for the localization of total Ror2 (Ror2mCherry, red channel) and Ror2 homodimers (splitYFP signal, green channel) in the absence (−Wnt5a) and presence (+Wnt5a) of co-injected Wnt5a and in the presence of 50 µg chlorpromazine. Ten optical sections of 1 µm thickness allowed us to visualize localization of the signals in the z-dimension. The white bar in the close-ups of the yz-plane marks the z-coordinate shown in the xy-images. (**b**) xy-planes at three different z-values illustrate the exclusively basal and basolateral localization of Ror2/Ror2 dimers in Wnt5a co-injected explants. (**c**) To quantify the z-distribution of the fluorescence signals, at least 11 explants (each with 4 to 6 different cells) derived from 3 independent experiments were analyzed. Individual fluorescence spots were selected in the maximum intensity projections, and the images were analyzed section by section for visibility of these spots. Shown is the fraction of fluorescence spots found in the xy-images as a function of the z-coordinate from basal (0 µm) to apical (9 µm). These data were calculated for each cell by dividing the number of fluorescence spots of a particular xy-plane by the number of spots in all planes. Shown are mean values and standard deviations from at least 11 explants. It is apparent that the BiFC signal (Ror2 homodimers) gets shifted to the basal side in the presence of Wnt5a; chlorpromazine and genistein revert this effect. (**d**) Paraffine sections of early gastrula stages revealed that Ror2 homodimers are located at the entire lateral site of animal cap cells in the absence of Wnt5a. In the presence of Wnt5a, however, the dimers are found exclusively at the basal side.

In animal caps, the BiFC signal of Ror2 homodimers displayed a similar localization as Ror2mCherry (Fig. 5a,c). Both, total Ror2 and Ror2 homodimers, are uniformly distributed in the entire plasma membrane. However, in animal caps of embryos co-injected with Wnt5a, the dimers disappeared from the apical surface and were located almost exclusively at the basolateral membrane. By contrast, Ror2mCherry remained more or less homogenously distributed (Fig. 5a–c). A slight shift of Ror2mCherry towards the basolateral membrane might reflect that mCherry labels all populations of Ror2, including Ror2 homodimers. Similar to Ror2 homodimers, Ror2/Fzd7 heterodimers also change their subcellular distribution. Again, they are found exclusively at the basal and basolateral membranes in Wnt5a injected animal caps (Supplementary Fig. 6a–c). We further confirmed the Wnt5a induced loss of apical Ror2 dimers by immunohistology on paraffin section of late blastula stage embryos (Fig. 5d).

This surprising result indicates that Ror2/Ror2 homodimers and Ror2/Fzd7 heterodimers form a subpopulation of membrane localized Ror2 molecules. Receptor dimers co-localize with monomeric Ror2 molecules, but only dimers disappear from the apical membranes in Wnt5a overexpressing explants. Thus, Wnt5a induces a symmetry break along the basal-apical axis; receptor dimers are found exclusively at the basolateral membrane. Together with the co-clustering of Wnt5a and Ror2^23^ and the decrease of the Ror2/dsh dimer fraction in the plasma membrane in response to Wnt5a, our data suggest, that a signalosome consisting of Wnt5a, Ror2, Fzd7 and dsh becomes internalized in response to Wnt5a binding to the receptor. Indeed, we were able to visualize internalization of Ror2 homodimers together with Ror2mcherry in Wnt5a treated AC explants (Supplementary Movie 2).

Based on our observations that both caveolin and clathrin dependent endocytosis regulate CE movements, and that Wnt5a signaling depends on caveolin-dependent endocytosis (this study) and clathrin dependent endocytosis^26,27^, we expected endocytosis inhibitors to block the Wnt5a-induced redistribution of Ror2 dimers. To test this, we added the endocytosis inhibitors genistein and chlorpromazine to the animal cap explants and analyzed the localization of Ror2 homodimers. Indeed, after blocking caveolin-dependent endocytosis, the Wnt5a effect was less pronounced (Fig. 5c and Supplementary Fig. 6a). In the absence of the inhibitor, two third of the BiFC spots were located in the basal-most 1-µm sheet. After blocking caveolin-dependent endocytosis, this basal localization was reduced to less than 50% (Fig. 5c). This change was highly significant (p < 0,05). Similarly, in the apical parts of the membrane, the frequency of the BiFC signal increased. The effect of the clathrin inhibitor chlorpromazine was even more pronounced (Fig. 5a–c). The BiFC signal was more or less equally distributed over the entire lateral membrane, similar to the localization in the absence of Wnt5a and similar to the distribution of Ror2 monomers (Fig. 5c).

Our data thus clearly reveal that Wnt5a induced redistribution of Ror2 homodimers is due to clathrin and caveolin dependent endocytosis of dimers at the apical membrane.

**Figure 6:**
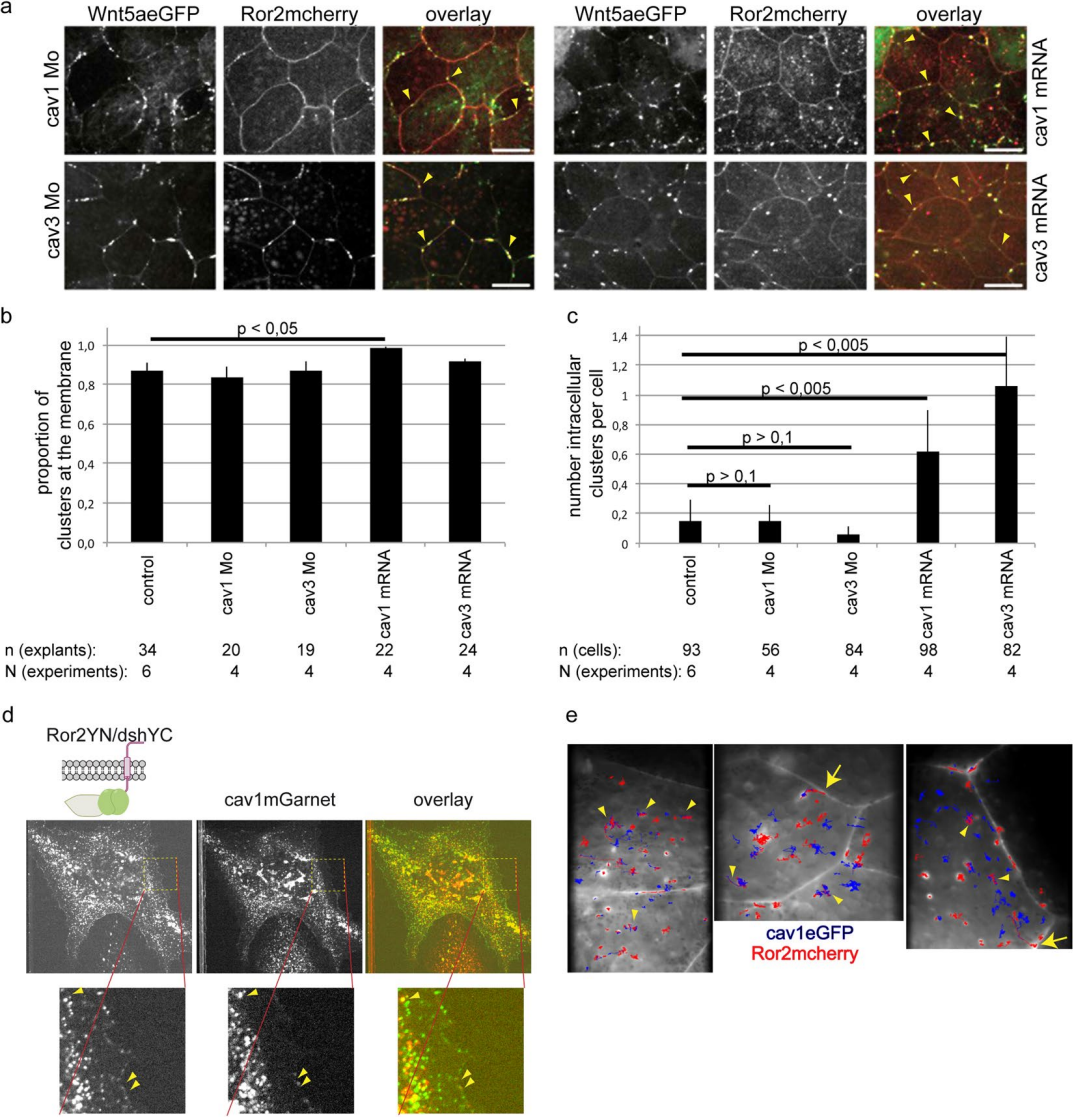
Caveolin regulates internalization of Wnt5a/Ror2 clusters. (**a**) Colocalization of Ror2mCherry/Wnt5aeGFP clusters in animal cap explants of caveolin1 and caveolin3 morphants and caveolin1 and caveolin3 overexpressing embryos. (**b**,**c**) Quantification of the clusters. More than 80% of the clusters are located at the plasma membrane. Overexpression of caveolin1 slightly increases the frequency of membrane localization. (**c**) Average number of intracellular clusters per cell. p values according to Students t-test. (**d**) The BiFC signal of Ror2/dsh dimers partially colocalizes with the red fluorescence of caveolin1mGarnet in transfected XTC-cells, both, at the plasma membrane and in the cytoplasm (arrowheads in the close-ups). (**e**) Single particle tracking using widefield fluorescence microscopy indicates that cav1eGFP (blue trajectory) and Ror2mcherry (red trajectory) co-migrate in the cytoplasm (arrowheads) and leave the membrane together and continue co-migrating in the cytoplasm (arrows).

We used the Ror2 clusters to enquire whether the Lef1 target genes of the caveolin family are involved in cluster formation and/or cluster internalization. Therefore, we counted clusters in the cytoplasm and plasma membranes of animal cap explant cells of caveolin morphants and caveolin overexpressing embryos (Fig. 6a). More than 80% of the clusters were found at the plasma membrane (Fig. 6b). In caveolin1 overexpressing embryos, this fraction was even as high as 98%. Similarly, the total number of clusters per cell increased after caveolin1 injection. In the cytoplasm, the number of Ror2/Wnt5a clusters is three- to sixfold greater in caveolin overexpressing explants (Fig. 6c). By contrast, we did not find any significant changes in caveolin morphants. This might be a consequence of the small number of intracellular Wnt5a/Ror2 clusters, here and also in wild-type embryos. In fact, they were present only in about one out of six cells. The slight increase of intracellular Ror2/Wnt5a clusters in caveolin overexpressing cells, however, might indicate that these clusters are internalized in a caveolin dependent manner. Indeed, we found Ror2/dsh dimers partially colocalized with caveolin1 in tissue culture cells (Fig. 6d). Widefield microscopy on live cells revealed colocalization of Ror2mcherry and caveolin1eGFP (Supplementary Movie 3). Trajectories of individual fluorescence spots demonstrate that caveolin1 and Ror2 comigrate within the cell and, more importantly, that Ror2 and caveolin1 comigrate from the membrane into the cell, indicating that they are internalized together (Fig. 6e).

## Discussion

**Figure 7:**
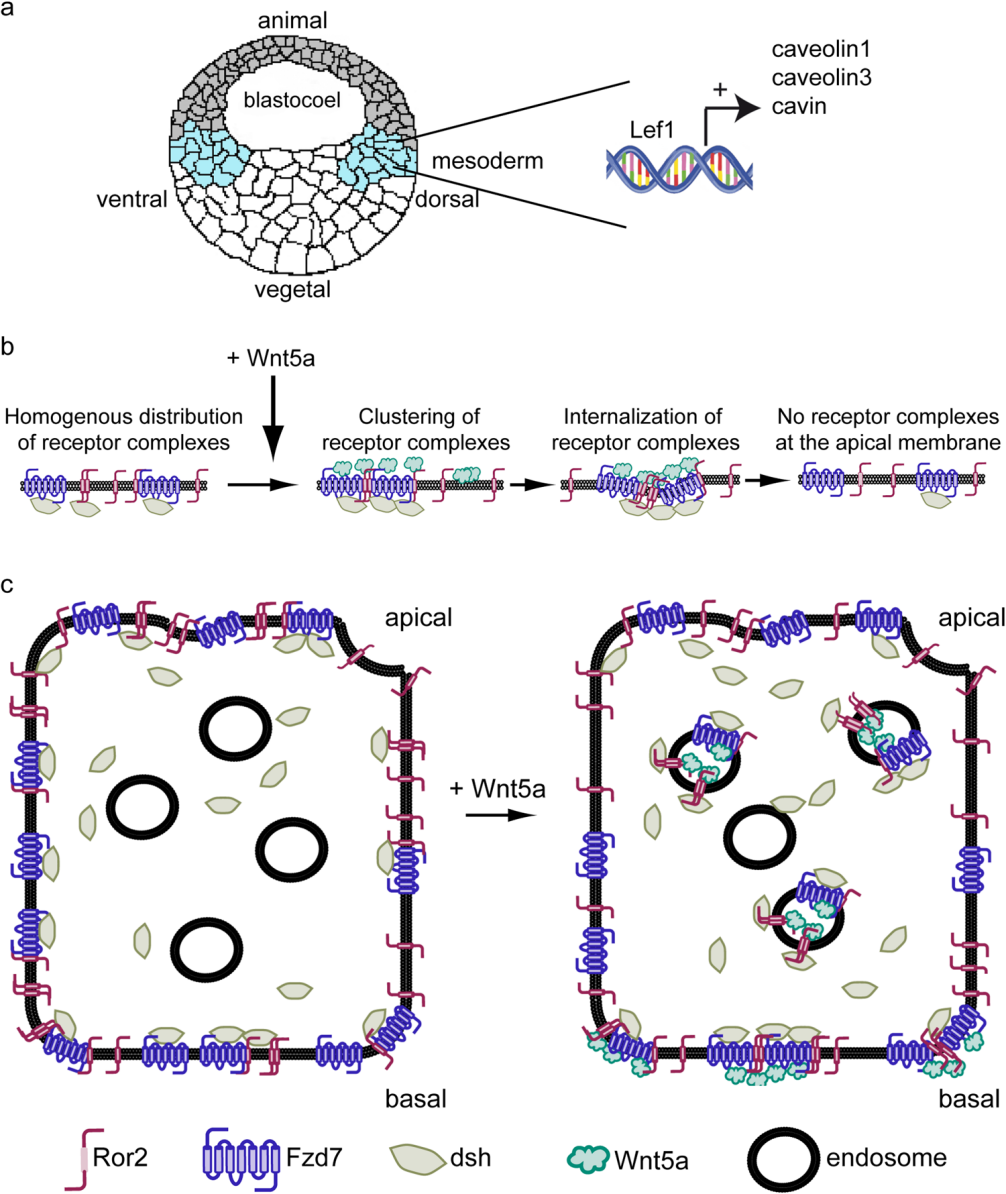
The Lef1 targets of the caveolin family are required for Wnt5a induced receptor complex internalization. (**a**) Lef1 regulates the expression of the caveolar core components caveolin1, caveolin3 and cavin1. (**b**) Wnt5a induces rapid clustering of non-canonical receptor complexes and subsequently their internalization via clathrin and caveolin dependend mechanisms, (**c**) Apical internalization of receptor clusters results in a symmetry break of polarized cells with membrane bound receptor clusters at the basal membrane.

The interplay between Lef1, caveolin dependent endocytosis and Wnt5a signaling shown here, lets us propose the following model (Fig. 7): At early stages Lef1 provides the mesoderm with the competence to react during gastrulation on Wnt5a in an appropriate manner. Relevant for this reaction is the expression of the caveolae core components caveolin1, caveolin3 and cavin1. In immediate response to Wnt5a, receptor complexes cluster at the cell surface. These complexes become asymmetrically internalized, resulting in Wnt5a/Ror2/Fzd/dsh located at the basolateral side of Wnt5a receiving cells.

In a microarray based transcriptome analysis we found *caveolin1*, *caveolin3* and *cavin1* among the TOP100 Lef1 regulated genes. All of them were downregulated in Lef1 morphants, indicating that the expression of caveolar core components depends on β-catenin/Lef1 regulated transcription. In general, the Wnt/β-catenin branch of the Wnt signaling network is important for the induction and positioning of the Spemann organizer^28^. The non-canonical Wnt branches activated by Wnt5a and Wnt11 regulate distinct aspects of CE movements in *Xenopus* in a non-redundant manner^24^. Wnt11 is necessary for polarization of mesodermal cells, Wnt5a for migration of polarized cells towards the midline^12^. This separation into different pathways is simplistic, however, because it ignores that the different Wnt signal transduction pathways including the Wnt/β-catenin, Wnt5a/Ror2 and Wnt11/PCP branches regulate each other at several levels of signal transduction^1^.

Herein, we have provided evidence that CE movements are regulated by clathrin and caveolin dependent endocytosis. We have further observed that Wnt5a induces clathrin and caveolin dependent endocytosis of Wnt5a signaling complexes and that endocytosis is necessary for Wnt5a/Ror2 signal transduction. Thus, in addition to inducing the Spemann organizer, early Wnt/β-catenin regulated gene expression is a prerequisite for trunk mesodermal cells to respond properly to non-canonical Wnt signaling during gastrulation. This does not necessarily mean that caveolins are direct Lef1 targets; instead, it might reflect that Lef1 regulates the cell fate/competence of early mesodermal tissue and that the downregulation of *caveolin1*, *caveolin3* and *cavin1* in Lef1 morphants is due to a change in cell fate/competence,

In mouse^29^ and *Xenopus*^30^, the expression of *caveolin* genes starts with the onset of gastrulation. In *Xenopus*, *caveolin* expression remains at low levels until neurula stages, with *caveolin1* and *cavin1* enriched in the notochord and *caveolin3* distributed in a salt and pepper pattern in multiciliated cells of the epidermis. Epistasis experiments revealed that caveolin1, caveolin3 and cavin1 cannot compensate the loss of Lef1. Thus, further Lef1 target genes are involved in regulating CE movements. One obvious candidate might be *Xnr-3*^8^. Our data demonstrates that caveolins and caveolin mediated endocytosis are involved in CE regulation. Thus, the low expression levels at early stages of development are of functional relevance. Treatment with endocytosis inhibitors revealed that caveolin and clathrin dependent endocytosis act in a redundant manner during *Xenopus* gastrulation. If this redundancy holds true for early embryogenesis of other vertebrates, it might explain why caveolin1/caveolin3 double knock-out mice are viable even though they lack caveolae^22^.

Based on the studies of Lee and Harland^31^, who showed that endocytosis is important for bottle cell formation at the dorsal blatoporus lip, we asked if endocytosis is also involved throughout gastulation. Indeed, blocking endocytosis with chlorpromazine and genistein indicates that endocytosis is necessary until the end of gastrula stages.

Fzd2 internalization after Wnt5a stimulation was shown to occur *via* clathrin mediated endocytosis^26^. Recently, Shojima and coworkers^32^ demonstrated that, for cancer cell activation, Wnt5a signals *via* endocytosis dependent and independent mechanisms. Using endocytosis inhibitors, we have shown here that Wnt5a dependent signaling in early embryogenesis needs internalization. Immunohistochemical analyses revealed that Wnt5aeGFP co-localizes with caveolin1 and early endosomal antigene (EEA), but not with clathrin^23^. In a proteomic screen, Zheng *et al*.^33^ identified Wnt5a among 148 caveolar proteins. Furthermore, Ror2, Fzd2 and Fzd5 co-precipitated with caveolin1^34^. Thus, evidence is accumulating that caveolin-dependent internalization plays a role for at least some branches of the non-canonical Wnt signaling network, in addition to clathrin-dependent internalization.

We focused our study on the Wnt5a/Ror2 pathway, because Wnt5a/Ror2 signaling is essential for CE movements^11^, Wnt5a/Ror2 signaling is not required for polarization of mesodermal cells early in gastrulation^12^ and Wnt5a/Ror2 signaling can be easily detected by fluorophore labelled Wnt5a/Ror2 clusters^23^. Most importantly, these clusters depend on Lef1 expression and thus are most likely regulated by Lef1 target genes. Furthermore, Ror2 clusters are specific towards Wnt5a because Wnt11, although inducing Fzd7 clusters in a similar manner as Wnt5a, hardly induces any Ror2 clusters (data not shown).

To characterize the composition of Ror2 clusters, we performed BiFC assays to demonstrate that Ror2 homo-dimerizes with Ror2 and hetero-dimerizes with Fzd7 and dsh. Since homodimers and heterodimers respond to Wnt5a in a similar manner, we suggest that the Wnt5a/Ror2 clusters observed in^23^ are Wnt5a induced multimeric protein complexes consisting of Wnt5a, Ror2, Fzd7 and dsh. These clusters are formed within a few minutes after adding Wnt5a protein, indicating that receptor clustering is an immediate response to Wnt5a. Interestingly, our BiFC experiments further revealed that Ror2 homodimers as well as Ror2/Fzd7 and Ror2/dsh heterodimers are formed also in the absence of Wnt5a, but cluster formation requires Wnt5a. Thus, in conflict with the model recently proposed by Curto *et al*.^35^, we provide evidence that Wnt5a is not necessary to form Ror2/dsh heterodimers, but instead induces or stabilizes multimeric ligand/receptor/adapter molecule complexes (Fig. 7). If CK1ε and p120 are activated by Wnt5a to form these multimeric complexes or if they provide an intracellular platform to stabilize Wnt5a induced multimeric complexes remains to be studied. Additionally, Wnt5a induces relocation of homo- and heterodimers. In general, the fraction of membrane located dimers decreases. This is most easily explained by internalization of multimeric Wnt5a/Ror2/Fzd7/dsh clusters. Indeed, overexpression of caveolin1 and caveolin3 increases the number of cytosolic clusters in animal cap explants and Ror2 and caveolin1 are internalized together and co-migrate in the cytoplasm. Thus, the reduction of cell surface located Ror2 in response to Wnt5a^35^ is most likely due to endocytosis. This might also explain why overexpression of caveolin can compensate for the loss of Wnt5a. Most likely, overexpressed caveolin facilitates clustering and internalization of the remaining Wnt5a receptor complexes in Wnt5a morphants.

Remarkably, the internalization of dimers/multimers was restricted to the apical site of animal cap explant cells. Caveolin dependent endocytosis is involved in this apical internalization of Ror2 clusters in addition to clathrin mediated endocytosis. Together with the observation that caveolin knock-down as well as drug-induced inhibition of endocytosis impairs gastrulation movements, our data revealed that Wnt5a dependent apical endocytosis of receptor complexes is important for Wnt5a/Ror2 signal transduction to regulate CE movements. Consistently, we found that the expression of the Wnt5a/Ror2 target genes *PAPC* and *pbk* is regulated by caveolin and caveolin dependent endocytosis.

Taken together, our results enable us to create a model describing how target genes of Lef1 interfere with Wnt5a/Ror2 signal transduction to regulate CE movements (Fig. 7). Lef1 regulates the expression of the caveolar core components caveolin1, caveolin3 and cavin1. This provides the cells with the competence to react appropriately to Wnt5a because these proteins are key parts of an endocytosis machinery which internalizes Wnt5a/Ror2/Fzd7/dsh clusters at the apical surface in response to Wnt5a binding. This internalization is essential for regulation of Wnt5a target genes and for proper CE movements. The mechanistic details of how internalization of apical Wnt5a/Ror2/Fzd7/dsh clusters elicits a cellular response remains to be analyzed, however. Our model is able to explain how Wnt5a promotes polarization of single epithelial IEC6 cells^36^. The asymmetrical distribution of Wnt5a/Ror2/Fzd7/dsh clusters at the basolateral membrane in response to Wnt5a exposure indicates a symmetry break in the apical/basal distribution of clustered, non-canonical Wnt pathway components. It remains to be clarified whether the remaining basolateral clusters indeed provide a signaling platform promoting migration, but the symmetry break of signaling components appears to be an attractive model of Wnt5a regulated cell migration. Recently, Yamamoto *et al*.^37^ observed that Wnt5a signaling was better stimulated at the basolateral side compared to the apical side in epithelial cells. If Wnt5a/Ror2/Fzd7/dsh clusters are internalized at the apical side and remain stable at the basolateral side also in these cells, the subcellular localization of non-canonical Wnt signalosomes might provide an explanation for this observation.

## Methods

All methods were carried out in accordance with relevant guidelines and regulations. All All experiments comply with the “Principles of Animal Care”. Permission for the experiments was given by the Regierungspräsidium Karlsruhe, *AZ* 35-9185.81/G-161/03.

### Constructs

Capped mRNAs were transcribed from linearized DNA templates using mMESSAGE mMACHINE (Ambion). Digoxigenin labeled antisense probes for *in situ* hybridization (ISH) were synthesized with DIG RNA labeling kit (Roche). Probes for ISH were *chordin*^38^, *PAPC*^11^, and the ORF of *caveolin1*, *caveolin3* and *cavin1* inserted into pGEMT. Templates for mRNA synthesis were: Lef∆HMG^39^, xLef1^40^, Wnt5aeGFP^23^, Ror2mCherry^41^, Fzd7YN^42^, Wnt5a^24^. The ORFs of caveolin1, caveolin3 and cavin1 were inserted in pCS2. Ror2YN, Ror2YC, Wnt11eGFP, Ror2∆CRDYC, Ror2∆CRDYN, Fzd7mCherry, caveolin1mGarnet and caveolin1eGFP were generated by fusion PCR and inserted into pCS2 (primer upon request). DshYC was kindly provided by Susanne Kühl (Ulm). Ror2∆CRD was a gift from Alexandra Schambony (Erlangen).

The following antisense morpholino oligonucleotides (Gene Tools, LaJolla) were used: Wnt5a^11^, Lef1^43^, caveolin1, caveolin3, cavin1 (this study, Supplementary Fig. 2).

### RNA isolation from DMZ explants and microarray

Microarray experiments to identify novel Lef1 target genes important for gastrulation were done exactly as described for the identification of Wnt5a and Wnt11 target genes^24^. The microarray analysis was performed on the *Xenopus* 4 × 44 K gene expression Chip (Agilent Technologies, Germany) by Atlas Biolabs (Berlin, Germany). Data sets are deposited on http://www.ncbi.nlm.nih.gov/geo/query/acc.cgi?acc=GSE84488. The TOP 100 spots of differentially expressed genes displayed more than 3.8-fold difference between morphants and control siblings at a significance level <0.05 (Supplementary Table 1).

### Cell culture

HEK293 cells were transfected by Calcium phosphate precipitation according to Gorman *et al*.^44^ with the reporter ATF2-Luciferase^45^, CMV-β-galactosidase and the indicated DNA constructs. 48 h after transfection luciferase activity was determined as described earlier^46^.

*Xenopus* tissue culture cells^47^ were transfected with TransPass transfection reagent (New England Biolabs) according to the instructions of the manufacturer. For live imaging cells were seeded 24 h after transfection on fibronectin coated chamber slides and cultivated for additional 24 h. Cells were analysed with spinning disc microscopy (Observer Z1, Zeiss) and Axiovision 4.8.2 software (Zeiss). Grey-value measurements of the subcellular distribution of fluorophore tagged proteins were carried out using ImageJ software (open source: http://rsbweb.nih.gov/ij/). Statistics of the grey-value distribution were done with Past3 software (open source: https://folk.uio.no/ohammer/past/).

### *Xenopus* embryos, micromanipulation and *in situ* hybridization

*Xenopus* embryos were obtained by *in vitro* fertilization and staged according to Nieuwkoop and Faber^48^. To target the axial mesoderm, both dorsal blastomeres of four-cell stage embryos were injected in the equatorial region. To target the animal cap, all four blastomeres of four-cell stage embryos were injected in the animal pole. To target one hemisphere of the developing embryo, one blastomere of two-cell stage embryos was injected in the animal hemisphere.

For RNA *in situ* hybridization (ISH) embryos were fixed for one hour in 4% formaldehyde at the indicated stages. ISH was performed as described^49^. Statistical analyses of the phenotypes were done with Χ^2^ test.

### RT PCR and realTime PCR

RNA was isolated from 5 AC explants by use of the TRIzol^®^ Plus RNA Purification Kit (Invitrogen, Carlsbad, California, USA). Prior to the isolation the AC explants were collected and stored in RNAlater (Ambion, Norwalk, CT, USA). After reverse transcription (M-MLV, Promega) cDNA was amplified according to standard protocols. Real time PCR was performed using iQ SYBR Green Supermix on an iCycler (BioRad, Hercules, CA, USA). Expression levels were calculated relative to *ornithine decarboxylase (ODC*) and normalized to wild-type explants. Results of four independent experiments were averaged, and statistical significance was calculated using Student’s *t*-test. Primer sequences and annealing temperatures are available upon request.

### DMZ and animal cap explants and live imaging

DMZ explants were dissected at stage 10.25 and cultivated in petri dishes coated with 1% BSA in 1x MBSH until their siblings reached stage 12.

Animal caps of stage 9 embryos were cut manually, placed on BSA coated glass cover slides and fixed with cover glass. To generate a cultivation chamber, the cover glass was separated from the cover slides by a thin layer of silicone. Recombinant Wnt5a protein (R&D Systems) was added by carefully changing MBSH in the cultivation chamber with MBSH containing 0,1 µg/µl Wnt5a and 0,1% BSA.

Time-lapse movies and z-stacks were captured using the Z1 Cell Observer Spinning Disc inverse microscope (Zeiss, Jena, Gemany). For the z-distribution of fluorophore-tagged proteins in animal cap explants ten optical sections with 1 µm distance were captured, starting from the basal side at position 0 µm, where cell protrutions were visible.

### Widefield microscopy and single-particle tracking

Images were acquired at room temperature on an inverted microscope (Axiovert 200, Zeiss) equipped with an oil immersion objective (alpha Plan-Apochromat 63×/1.46 Oil, Zeiss). mCherry was excited by a solid state laser with wavelength 561 nm (Gem 561, Laser Quantum, Konstanz, Germany) by an illumination power of 2 W/cm². The laser beam was guided via dichroic mirrors (AHF, Tübingen, Germany) to an AOTF (AOTFnC-400.650, AA Opto-Electronic, Orsay Cedex, France) for control of the laser intensities at the sample. After passing through the excitation dichroic (z 405/473/561/635, AHF, Tübingen, Germany), the emission light was split into two color channels by a dichroic beam splitter (Optosplit, Cairn Research Ltd, UK), filtered by 525/50 nm (center wavelength/width) and 607/70 nm filters for the eGFP-mCherry pair, and 525/45 nm and 641/75 nm filters for the YFP-mCherry pair (all BrightLine® single-band bandpass filters, Semrock, New York, NY). The two color-filtered images were focused side-by-side on an electron-multiplying CCD camera (Ixon Ultra 897, Andor, Belfast, UK) set to an exposure time of 50 ms.

Single particle tracking was performed using a livePALM software^50^ and a particle tracking program provided by John C. Crocker and colleagues (http://www.physics.emory.edu/faculty/weeks//idl/). Trajectories were acquired over 1000–2000 frames. Only events with photon numbers higher than 1000 per frame were registered. Particles in adjacent frames were linked if their displacement between successive frames was less than 2.5 µm. To generate time lapse movies, stacks were analyzed using Fiji software^51^. Bleaching was compensated using Fiji software, and the SPIM registration plugin was used for drift compensation^52^.

## Supplementary information

Supplementary Information

Suoolementary Info File '1

Supplementary Figures

Supplementary movies
