# Supplementary material for: Lef1 regulates caveolin expression and caveolin dependent endocytosis, a process necessary for Wnt5a/Ror2 signaling during Xenopus gastrulation
Source: Sci Rep. 2019 Oct 30;9:15645. doi: 10.1038/s41598-019-52218-1 (PMC6821757; doi:10.1038/s41598-019-52218-1)
Supplement: Supplementary file 1 — Supplementary Information [file 41598_2019_52218_MOESM1_ESM.docx]

**Lef1 regulates caveolin expression and caveolin dependent endocytosis, a process necessary for Wnt5a/Ror2 signaling during *Xenopus* gastrulation**

Katharina Puzik*^1^, Veronika Tonnier*^1^, Isabell Opper^1^, Antonia Eckert^2^, Lu Zhou^2^, Marie-Claire Kratzer^1^, Ferdinand le Noble^1,3^, Gerd Ulrich Nienhaus^2,3,4,5^, Dietmar Gradl^1#^

*Equal contribution

^#^ Corresponding author

KP: katharina.puzik@kit.edu

VT: veronika.tonnier@gmx.de

IO: isabel.opper@googlemail.com

AE: antonia.eckert@kit.edu

LZ: lu.zhou@kit.edu

MCK: kratzer.marieclaire@gmx.de

FLN: ferdinand.noble@kit.edu

GUN: uli@uiuc.edu

DG: dietmar.gradl@kit.edu

Affiliations:

*^1^Department of Cell and Developmental Biology, Karlsruhe Institute of Technology, 76128 Karlsruhe, Germany*

*^2^Institute of Applied Physics, Karlsruhe Institute of Technology, 76128 Karlsruhe, Germany*

*^3^Institute of Toxicology and Genetics, Karlsruhe Institute of Technology, 76344 Eggenstein-Leopoldshafen, Germany*

*^4^Institute of Nanotechnology, Karlsruhe Institute of Technology, 76344 Eggenstein-Leopoldshafen, Germany*

*^5^Department of Physics, University of Illinois at Urbana-Champaign, Urbana, Illinois 61801, USA*

**Supplementary Figure** **1** a) Both dorsal blastomeres of *Xenopus* 4-cell stage embryos were injected with either 4 pmol of the control morpholino (control) or the xLef1specific morpholinos (LefMo). DMZ cells were explanted at the onset of gastrulation and mRNA was isolated when siblings reached late gastrulation (stage 12). Purified mRNA probes (RIN > 8) where analyzed on an ATLAS Microarray. The TOP-100 of the differentially regulated genes include the caveolar core components caveolin1 (cav1), caveolin3 (cav3) and cavin1. The total list of the TOP-100 target genes is found in Supplementary Table 1. b) Expression of the caveolar core components during early *Xenopus* development revealed that cavin1 and caveolin1 (cav1) are co-expressed in the notochord from early tailbud stage on. Caveolin3 (cav3), instead, is expressed in a salt and pepper pattern in multiciliated cells of the epidermis. At gastrula stages, no local accumulation of the caveolin3 mRNA was detectable.

c) Lef1 (LefMo) were injected into one blastomere of *Xenopus* two-cell stage embryos. The asterisks mark the injected site. *In situ* hybridization revealed that 10 out of 29 of the Lef1 morphants embryos displayed markedly reduced expression of caveolin3 in muticiliated cells of the skin. In Lef1 morphant embryos co-injected with XLef1 mRNA (500 pg) only 2 out of 20 embryos showed reduced cav3 expression.

**Supplementary Table 1**

TOP 100 list of the differentially regulated genes in Lef1 morphants. Caveolin1, caveolin3 and cavin1 are highlighted in red.

**Supplementary Figure** **2** **Caveolin1, caveolin3 and cavin1 are necessary for convergent extension movements**. a) Sequences of caveolin1 (cav1), caveolin3 (cav3) and cavin1 morpholinos and their target mRNAs. The AUG start codon is indicated in bold. b) Dose response curve of the phenotypes to increasing amounts of the caveolin3, caveolin1 and cavin1 specific antisense morpholinos indicates that the severity of chordin mislocalization increases with the morpholino dose. c) Cavin1 and caveolin1 patially restore chordin expression in cavin1 morphants. 1 pmol cavin1 Mo was coinjected with 500 pg cavin1 and caveolin1 mRNA, respectively. p values according to χ^2^ test. d) In transfected HEK293 cells, caveolin1 and caveolin3 significantly activate the non-canonical Wnt reporter ATF2Luc. e) However, in Wnt5a stimulated cells (co-transfected with Wnt5aeGFP), caveolin1 and caveolin3 have no effect. p values of at least nine independent transfections were determined according to Student’s t-test. f) Lef1 specific morpholinos were injected into the dorsal marginal zone of 4-cell stage embryos and co-injected with caveolin1 mRNA and a cocktail of caveolin1, caveolin3 and cavin1 mRNA (50 pg each) and analyzed at stage 14 for the localization of the axial mesodermal marker gene *chordin*. n.s. not significant according to χ^2^ test.

**Supplementary Figure** **3 Endocytosis is necessary for convergent extension movements**. a) To determine the time-window where caveolin-dependent endocytosis is relevant for gastrulation, we incubated the embryos with 150 µM genistein for different periods from late blastula (stage 9) until early neurula (stage 14) and analyzed the localization of *chordin* expression and the defects in blastopore closure. Quantification of blastopore defects (b) and *chordin* localization (c) revealed that the longer the inhibitor is present, the more severe are the gastrulation defects. Significant effects were only observed when the inhibitor was added at stage 9. p-values according to χ^2^ test, n: number of embryos. d) RT-PCR revealed that the caveolae-specific endocytosis inhibitor genistein activates the expression of the Wnt5a target gene *pbk* in transfected XTC cells. Shown are mean values and SEM from three independent experiments. p-values according to Student’s t-test.

**Supplementary Movie 1** Time lapse movie to Supplementary Figure 4.

**Supplementary Figure** **4** a) Drift corrected snapshots of Supplementary Movie 1. Scale bar, 5 µm. b) The yellow region of interest (ROI) of frame 120 s was analyzed using Fiji software. A segmented line with a width of 2 pixels was drawn along the membrane, and the intensity profile was fitted with a superposition of 5 Gaussian functions representing the 5 intensity peaks indicated by numbers in the ROI. The fitted widths (full widths at half maximum, FWHM) are shown in the table on the right.

**Supplementary Figure** **5 BiFC assay** a) Bimolecular fluorescence complementation (BiFC) assay on animal cap explants revealed that Ror2 forms homodimers and heterodimerizes with Fzd7. The N-terminus and C-terminus of YFP (YN and YC) fused to Ror2 or Fzd7 do not form a fluorescent protein. However, co-injection of YN and YC –tagged receptor molecules resulted in the constitution of a fluorescent protein located in the membrane, indicating that the receptor molecules form dimers. Homodimerization of Ror2 depends on the extracellular CRD region because the splitYFP constructs fused to deletion mutant Ror2∆CRD fail to reconstitute a fluorescent protein. b) Median and dispersion (25 – 75 % range) are shown as box-and-whiskers plots to illustrate the membrane localization of total Ror2 as well as Ror2/Ror2 and Ror2/dsh dimers in the absence and presence of Wnt5a. While receptor dimers are less prominently found at the membrane in the presence of Wnt5a, the localization of total Ror2 remains almost unchanged. c) Box-and-whiskers plots show that the effect of Wnt5a is partially reverted in the presence of endocytosis inhibitors. The blue dashed line indicates the median of Ror2/dsh in the absence of Wnt5a, the red dashed line in the presence of Wnt5a.

**Supplementary Figure** **6 Wnt5a induces disappearance of Fzd7/Ror2 dimers at the apical membrane.** a) Animal cap explants were analyzed for localization of total Ror2 (Ror2mCherry, red channel), Ror2 homodimers (splitYFP signal, green channel) and Fzd7/Ror2 dimers (splitYFP signal, green channel) in the absence (–Wnt5a) and presence (+Wnt5a) of co-injected Wnt5a and in the presence of 50 µM genistein. Ten optical sections of 1 µm thickness allowed us to visualize localization of the signals in the z-dimension. The white bar in the close-ups of the yz-plane marks the z-coordinate shown in the xy-images. b) Selected xy-planes at three different z-values illustrate the exclusively basal and baso-lateral localization of Fzd7/Ror2 dimers in Wnt5a co-injected explants. c) Quantification of the z-distribution of the fluorescence signals, as described in the legend of Figure 5. A total of more than 3,500 splitYFP fluorescence spots and more than 4500 mCherry fluorescence spots were counted in optical planes of 14 explants derived from four independent experiments. Shown is the fraction of fluorescence spots in the xy-plane as a function of the z-coordinate, from basal (0 µm) to apical (9 µm). Obviously, in the presence of Wnt5a, the splitYFP signal (Fzd7/Ror2) is shifted to the basal side.

**Supplementary Movie** **2** Time lapse movies of animal explants incubated 15 min before imaging with 0.1 µg/µl Wnt5a revealed that both, Ror2 homodimers (left) and Ror2mcherry (right) are internalized as huge receptor complex. Scale bar 2 µm.

**Supplementary Movie** **3** Co-migration cav1eGFP and Ror2mcherry in animal cap explants. 200 pg cav1eGFP were co-injected with 200 pg Ror2mcherry and 200 pg Wnt5a mRNA. Scale bar 1µm.
