# Supplementary material for: Lef1 regulates caveolin expression and caveolin dependent endocytosis, a process necessary for Wnt5a/Ror2 signaling during Xenopus gastrulation
Source: Sci Rep. 2019 Oct 30;9:15645. doi: 10.1038/s41598-019-52218-1 (PMC6821757; doi:10.1038/s41598-019-52218-1)
Supplement: Supplementary file 3 — Supplementary Figures [file 41598_2019_52218_MOESM3_ESM.pdf]

Lef1 regulates caveolin expression and caveolin dependent endocytosis, a process necessary for Wnt5a/Ror2 signaling during *Xenopus* gastrulation

Katharina Puzik*[1], Veronika Tonnier*[1], Isabell Opper[1], Antonia Eckert[2], Lu Zhou[2], Marie-Claire Kratzer[1], Ferdinand le Noble[1,3], Gerd Ulrich Nienhaus[2,3,4,5], Dietmar Gradl[1#]

*Equal contribution

[#] Corresponding author

KP: katharina.puzik@kit.edu

VT: veronika.tonnier@gmx.de

IO: isabel.opper@googlemail.com

AE: antonia.eckert@kit.edu

LZ: lu.zhou@kit.edu

MCK: kratzer.marieclaire@gmx.de

FLN: ferdinand.noble@kit.edu

GUN: uli@uiuc.edu

DG: dietmar.gradl@kit.edu

Affiliations:

[1]*Department of Cell and Developmental Biology, Karlsruhe Institute of Technology, 76128 Karlsruhe, Germany*

[2]*Institute of Applied Physics, Karlsruhe Institute of Technology, 76128 Karlsruhe, Germany*

[3]*Institute of Toxicology and Genetics, Karlsruhe Institute of Technology, 76344 Eggenstein-Leopoldshafen, Germany*

[4]*Institute of Nanotechnology, Karlsruhe Institute of Technology, 76344 Eggenstein-Leopoldshafen, Germany*

[5]*Department of Physics, University of Illinois at Urbana-Champaign, Urbana, Illinois 61801, USA*

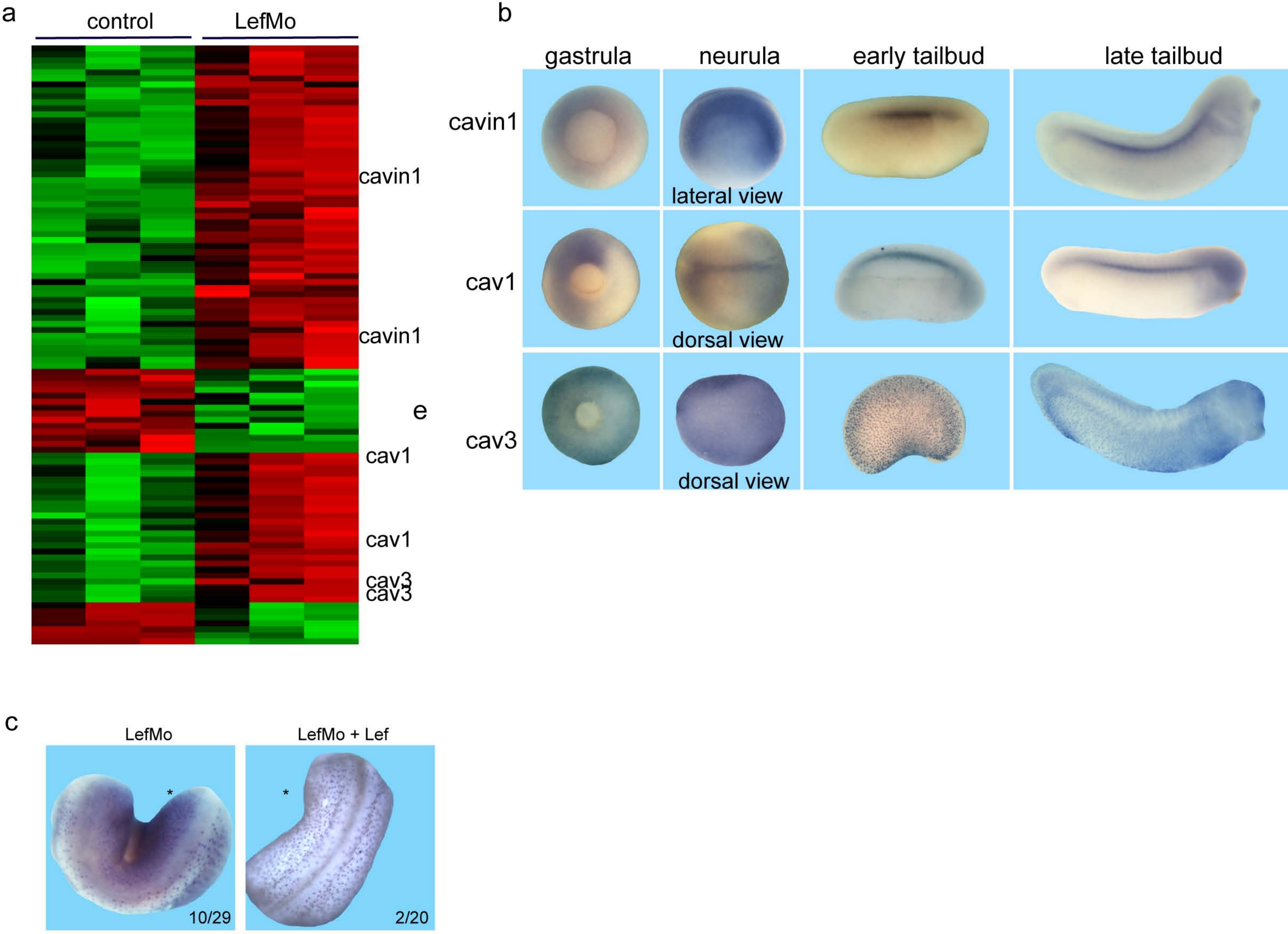

Puzik et al., Supplementary Figure 1

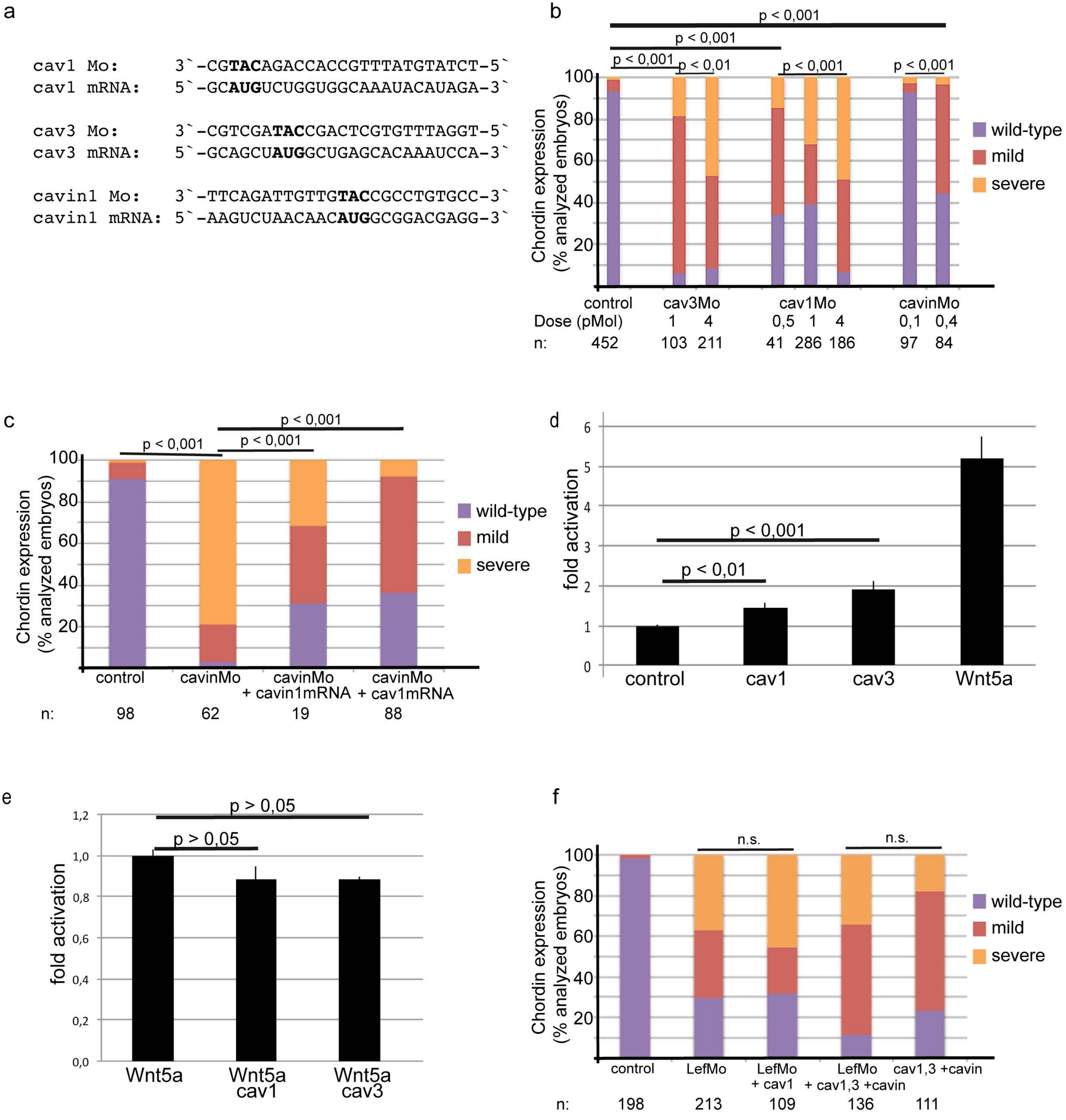

Puzik et al., Supplementary Figure 2

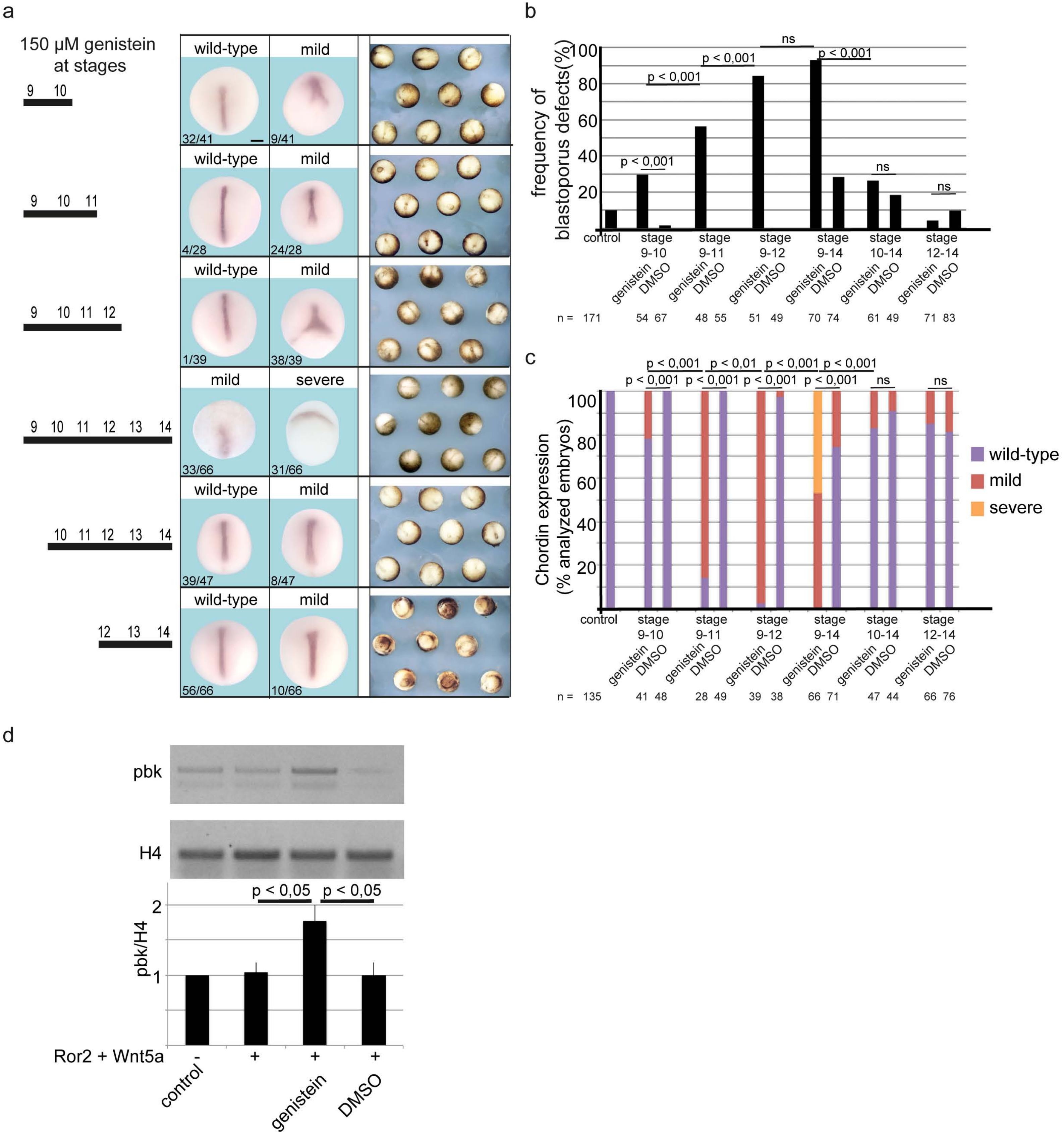

Puzik et al., Supplementary Figure 3

a

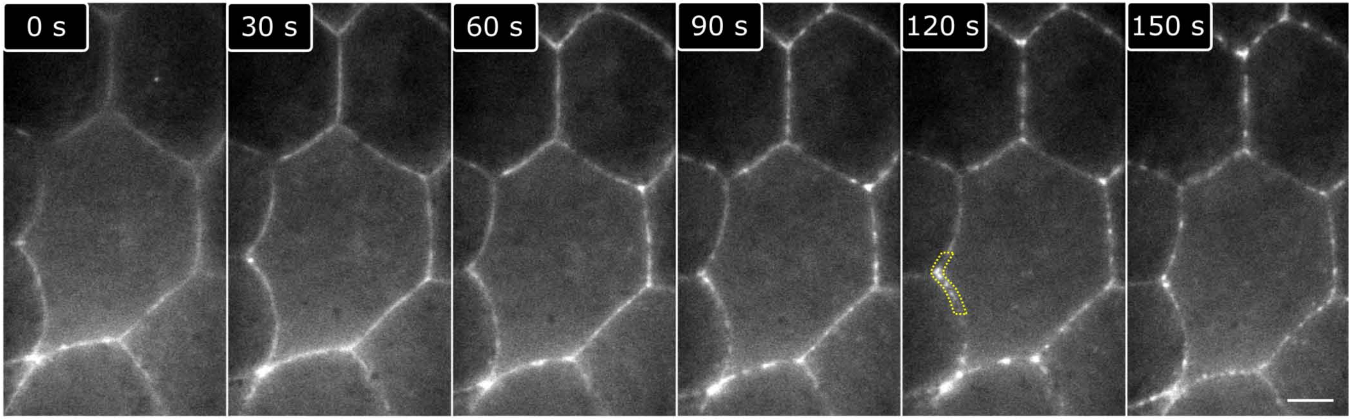

b

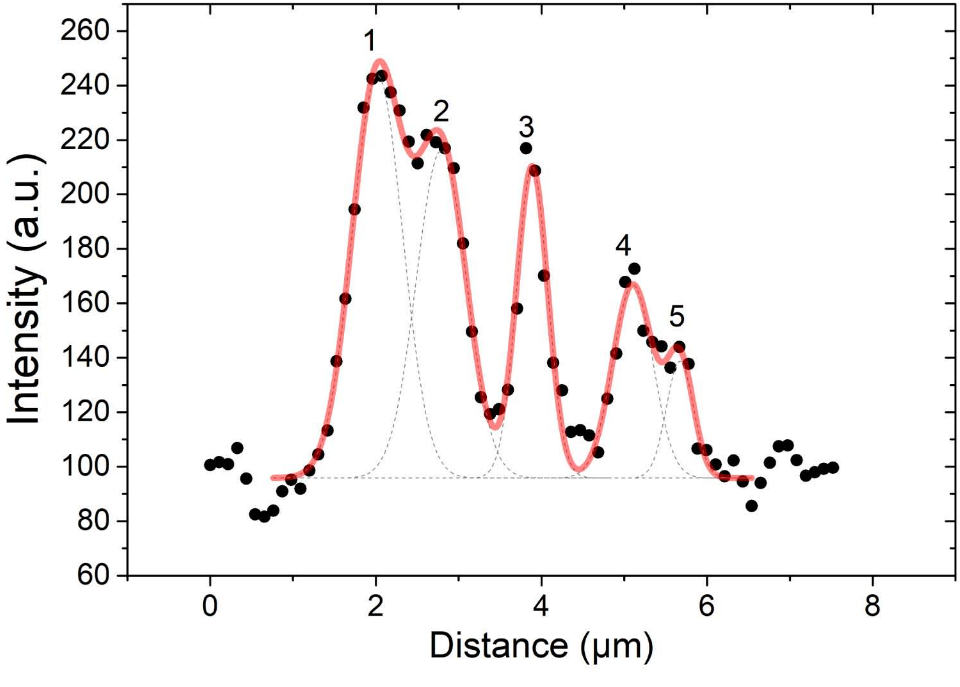

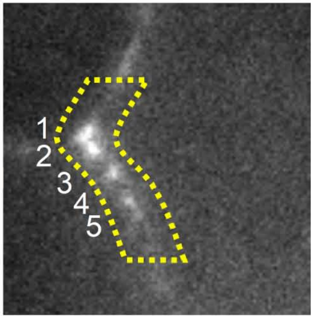

Fit No.	FWHM
1	(720 ± 50) nm
2	(710 ± 60) nm
3	(442 ± 27) nm
4	(570 ± 80) nm
5	(390 ± 90) nm

Puzik et al., Supplementary Figure 4

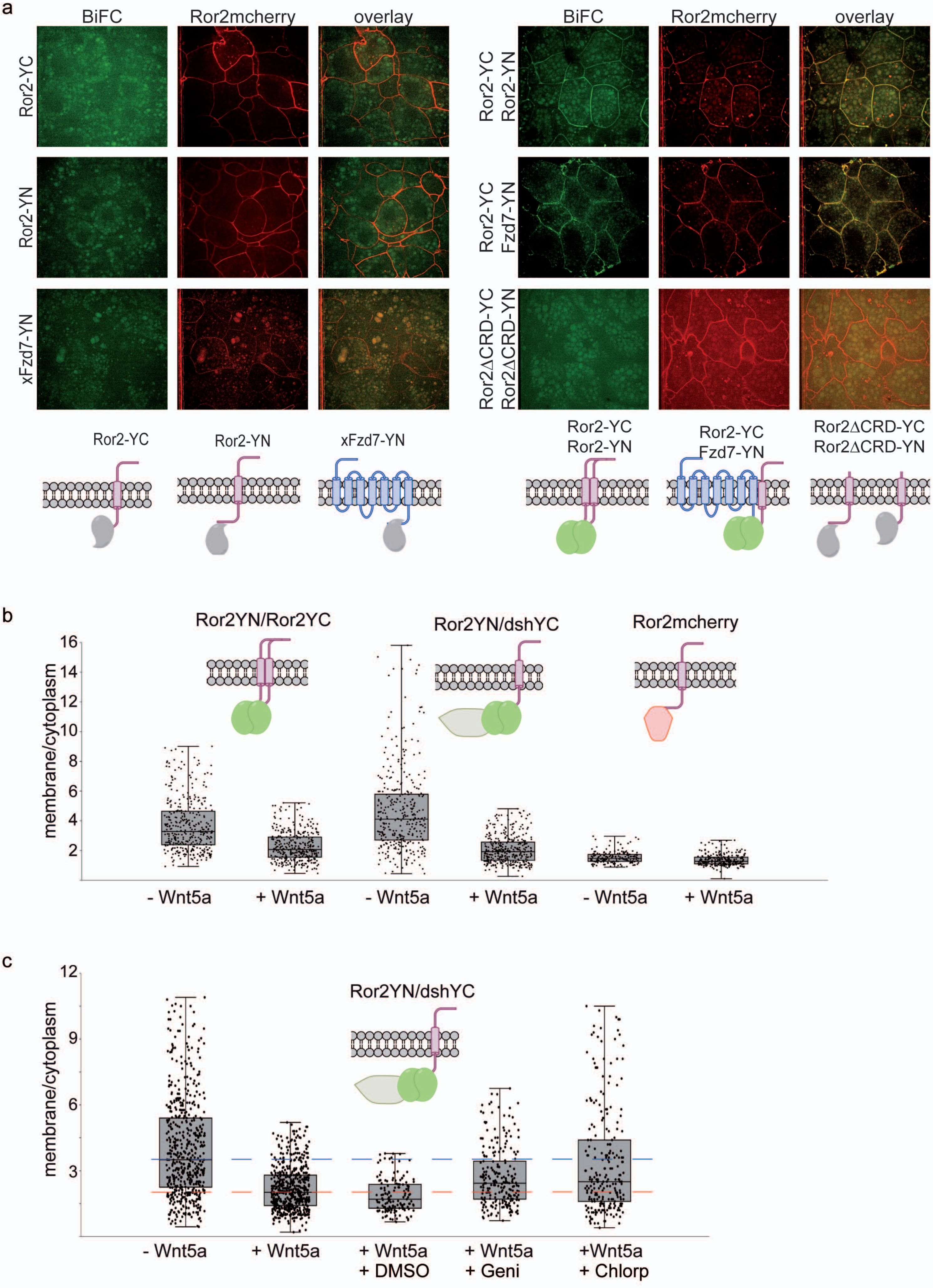

Puzik et al. Supplementary Figure 5

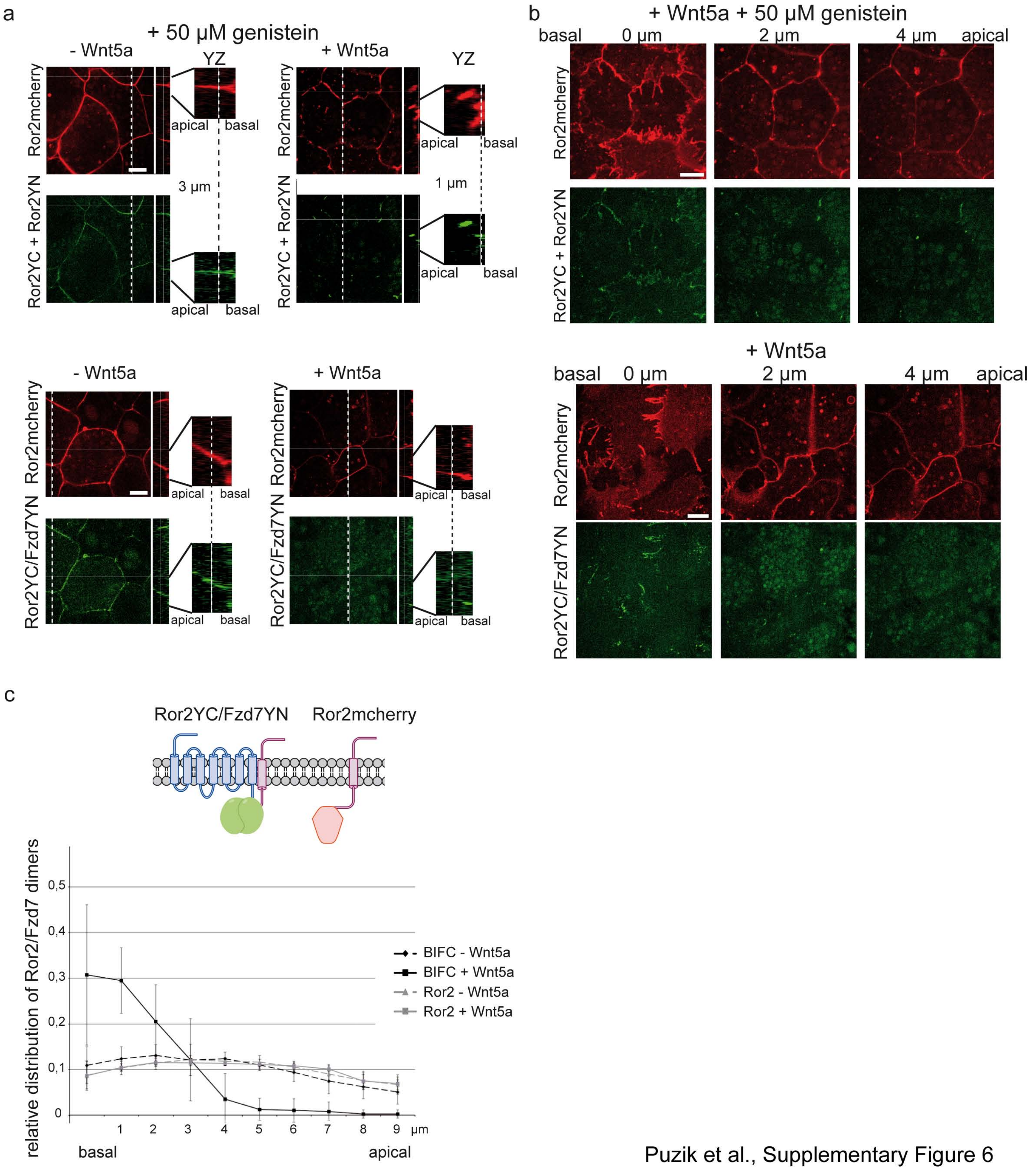

Puzik et al., Supplementary Figure 6
